# Structure and Biological Properties of Ribosome-Inactivating Proteins and Lectins from Elder (*Sambucus nigra* L.) Leaves

**DOI:** 10.3390/toxins14090611

**Published:** 2022-09-01

**Authors:** Rosario Iglesias, Rosita Russo, Nicola Landi, Mariangela Valletta, Angela Chambery, Antimo Di Maro, Andrea Bolognesi, José M. Ferreras, Lucía Citores

**Affiliations:** 1 Department of Biochemistry and Molecular Biology and Physiology, Faculty of Sciences, University of Valladolid, E-47011 Valladolid, Spain; 2 Department of Environmental, Biological and Pharmaceutical Sciences and Technologies (DiSTABiF), University of Campania ‘Luigi Vanvitelli’, Via Vivaldi 43, 81100 Caserta, Italy; 3 Department of Experimental, Diagnostic and Specialty Medicine-DIMES, Alma Mater Studiorum-University of Bologna, Via S. Giacomo 14, 40126 Bologna, Italy

**Keywords:** anticancer agents, galactose, lectin, nanoLC–tandem mass spectrometry (nLC-MS/MS), protein synthesis (inhibition), ribosome-inactivating protein (RIP), ricin, sugar binding

## Abstract

Ribosome-inactivating proteins (RIPs) are a group of proteins with rRNA N-glycosylase activity that catalyze the removal of a specific adenine located in the sarcin–ricin loop of the large ribosomal RNA, which leads to the irreversible inhibition of protein synthesis and, consequently, cell death. The case of elderberry (*Sambucus nigra* L.) is unique, since more than 20 RIPs and related lectins have been isolated and characterized from the flowers, seeds, fruits, and bark of this plant. However, these kinds of proteins have never been isolated from elderberry leaves. In this work, we have purified RIPs and lectins from the leaves of this shrub, studying their main physicochemical characteristics, sequences, and biological properties. In elderberry leaves, we found one type 2 RIP and two related lectins that are specific for galactose, four type 2 RIPs that fail to agglutinate erythrocytes, and one type 1 RIP. Several of these proteins are homologous to others found elsewhere in the plant. The diversity of RIPs and lectins in the different elderberry tissues, and the different biological activities of these proteins, which have a high degree of homology with each other, constitute an excellent source of proteins that are of great interest in diagnostics, experimental therapy, and agriculture.

## 1. Introduction

Ribosome-inactivating proteins (RIPs) are a group of proteins with rRNA N-glycosylase activity (EC 3.2.2.22) that catalyze the elimination of a specific adenine located in the sarcin–ricin loop (SRL) present in the large rRNA of eukaryotes and prokaryotes [1,2]. The elimination of this adenine (A4324 in rat ribosomes, or the equivalent in other organisms) inactivates ribosomes, which leads to the irreversible inhibition of protein synthesis and, therefore, cell death [1,2]. RIPs have been classified according to their structure as type 1 RIPs, consisting of a polypeptide chain with N-glycosylase activity, and type 2 RIPs, formed by two polypeptide chains, an A (active) chain with enzymatic activity, and a B (binding) chain with lectin activity that can bind to receptors on the surface of cells, facilitating the entry of RIP [2]. Some type 2 RIPs, such as ricin, are extremely toxic, while others have low toxicity; this is because the binding of the B chain to oligosaccharides present on the surface of cells is less effective, and because once internalized, the RIP follows an intracellular pathway different from ricin [1,3,4]. The toxicity of type 1 RIPs is lower, as they lack the lectin part and are, therefore, unable to bind to cells as type 2 RIPs do. Although the structure, activity, and mode of action of RIPs are known, their biological function is unclear. It has been proposed that these proteins could play an important role in the defense of plants against viruses, fungi, and insects [5,6].

Because of their diverse activities, RIPs, alone or as part of a conjugate, are good candidates for developing selective antiviral and anticancer agents [1,6]. Conjugates consist of a component directed against the target, such as an antibody, lectin, or growth factor, attached to a toxic component. RIPs have been used as the toxic component in several conjugates that have been tested in experimental therapies against various malignancies [3,7]. In agriculture, RIPs have been shown to increase resistance against viruses, fungi, and insects in transgenic plants [5,6].

RIPs are present in many angiosperm plants, both monocotyledonous and dicotyledonous, although in some plant families it is more common to find RIPs than in others; therefore, there are families such as Poaceae, Euphorbiaceae, Cucurbitaceae, Caryophyllaceae, Amaranthaceae, and Phytolaccaceae where several species with RIPs have been found, and other families where they have never been found [2]. Some species contain a wide variety of diverse RIPs, such as rice [8], or species of the genus *Phytolacca* [9,10].

The case of the genus *Sambucus* is unique, since more than 40 RIPs and lectins have been isolated and characterized to varying degrees from species belonging to this taxon [3,11]. Type 2 RIPs isolated from *Sambucus* have the peculiarity that, although they are enzymatically more active than ricin, they lack the high toxicity of ricin to cells and animals [11]. The presence in the same species of type 2 RIPs (heterodimeric and tetrameric), lectins (monomeric and homodimeric) structurally related to the above, together with type 1 RIPs, make the genus *Sambucus* an ideal model with which to study these proteins. Although these kinds of proteins can be found in other species, most of them have been obtained from elderberry (*Sambucus nigra* L.). Type 1 RIPs, type 2 RIPs (heterodimeric and tetrameric), and B-chain-related lectins have been obtained from the bark, seeds, flowers, and fruits, but the proteins from the leaves of this species have never been isolated. In this work we have isolated and characterized the most abundant RIPs and lectins in elderberry leaves by investigating their main physicochemical and structural properties, including amino acid sequence. We further studied their most important biological properties, and through in silico experiments, we explored potential mechanisms of sugar binding to these proteins.

## 2. Results

### 2.1. Isolation of RIPs and Lectins from Elderberry Leaves

**Figure 1:**
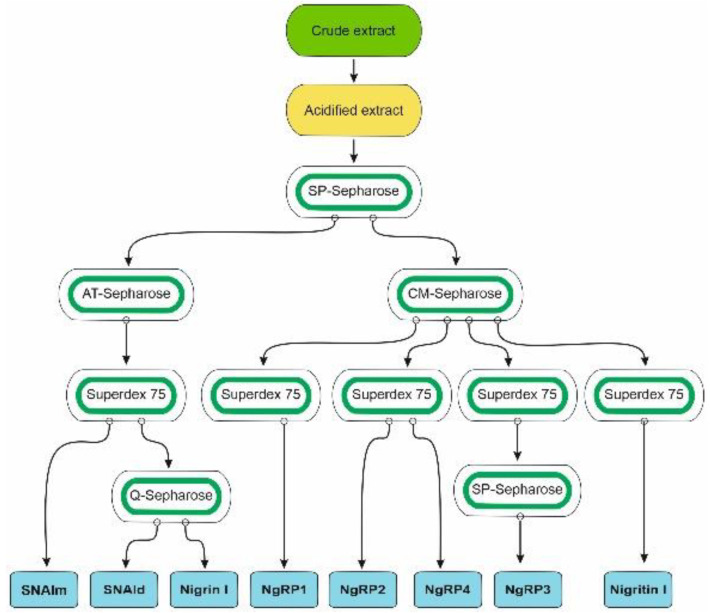
Schematic overview of the procedures used to purify the RIPs and lectins from *S. nigra* leaves. All steps of purification are detailed in the Materials and Methods.

Species of the genus *Sambucus* are one of the best sources for the isolation of RIPs and related lectins. These proteins have been found in *Sambucus ebulus* L. (dwarf elder), *S. nigra* L. (European elder), *S. sieboldiana* (Miq.) Blume ex Schwer. (Japanese elder), and *S. racemosa* L. (red elder) [3,11]. The most frequently used species, and the one with the greatest variety of these proteins, is *S. nigra*, in which RIPs and lectins have been isolated and characterized from bark, fruits, seeds, flowers, and pollen. Although RIPs from leaves of this species have not yet been isolated, in data banks the sequences of three proteins obtained from *S. nigra* leaves cDNA can be found: a type 2 RIP with an amino acid sequence similar to that of nigrin b from bark (named nigrin l), a monomeric lectin with a sequence similar to that of SNAIV from fruits (named SNAlm), and a homodimeric lectin with a sequence resembling that of SELld from the leaves of *S. ebulus* (named SNAld). Therefore, we aimed to isolate and characterize the RIPs and lectins from elderberry leaves. For this purpose, we optimized a standard RIP purification procedure [12] previously used to isolate RIPs and lectins from elderberry bark [13] and dwarf elder leaves [14]. A schematic overview of the procedures used to purify the RIPs and lectins from *S. nigra* leaves is shown in Figure 1.

A great difficulty in carrying out this purpose is the extraordinary variability that these proteins present in the leaves. For this reason, two purifications were derived from the leaves of *S. nigra*. Two crude extracts were prepared from 700 and 540 g of leaves. Then, an acidified crude extract was obtained and subjected to ion exchange SP-Sepharose chromatography. After washing the column with sodium acetate (pH 4.5), the bound protein was eluted first with 5 mM sodium phosphate (pH 6.66), and then with sodium chloride. The fraction eluted with sodium phosphate displayed both protein synthesis inhibitory activity and erythrocyte agglutination ability, while the fraction eluted with sodium chloride inhibited protein synthesis but failed to agglutinate erythrocytes (data not shown). The yield of proteins was different in the two preparations: in the first, a higher amount of protein was obtained by eluting with sodium phosphate (1.4-fold higher); in the second, a better yield was obtained by eluting with sodium chloride (threefold higher). Therefore, we used the first preparation for purifying proteins eluted with sodium phosphate and the second preparation for purifying proteins eluted with NaCl.

**Figure 2:**
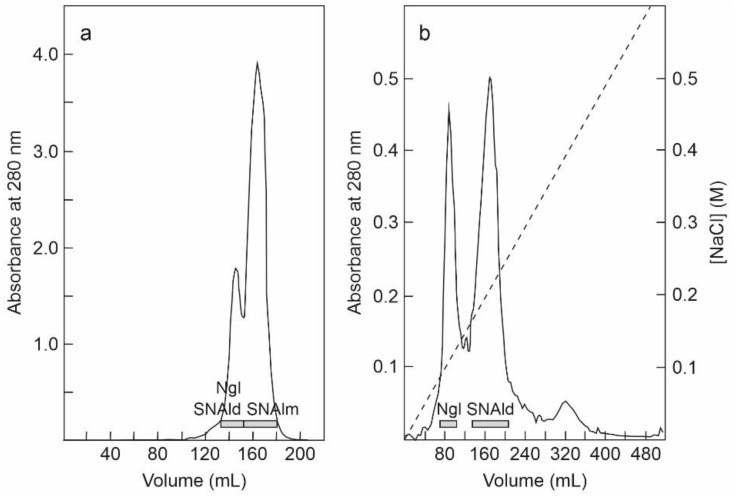
Purification of nigrin l, SNAlm, and SNAld: (**a**) The fraction eluted with lactose from the AT-Sepharose column was concentrated and chromatographed using Superdex 75 HiLoad. The fractions of the first peak contained nigrin l (Ngl) and SNAld, and those of the second peak contained SNAlm (horizontal bars); (**b**) the fractions of the first peak shown on panel (**a**) were dialyzed and subjected to chromatography using Q-Sepharose that was eluted with an NaCl gradient (dashed line), as indicated in the Materials and Methods. The fractions of the first peak contained nigrin l (Ngl) and those of the second peak contained SNAld (horizontal bars).

The fraction eluted with sodium phosphate from the SP-Sepharose column was further purified by affinity chromatography using an acid-treated (AT)-Sepharose column. After washing with buffer, the D-galactose-binding proteins were eluted from the column with 0.2 M lactose, concentrated, and subjected to chromatography using a Superdex 75 HiLoad column (Figure 2a). The first peak contained nigrin l and SNAld, and the second contained SNAlm. The fractions of the first peak were dialyzed and subjected to Q-Sepharose chromatography. Proteins were eluted from the column with an NaCl gradient yielding two peaks (Figure 2b). The fractions of the first peak contained nigrin l and those of the second peak contained SNAld. The estimated yields of this preparation were 2, 12, and 1.4 mg per 100 g of leaves for nigrin l, SNAlm, and SNAld, respectively.

**Figure 3:**
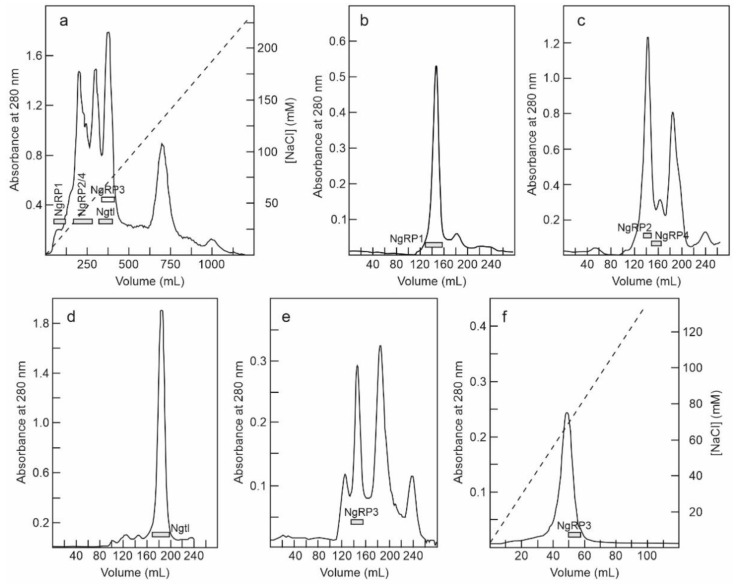
Purification of nigrin-Related Proteins 1–4, and nigritin l: (**a**) The proteins eluted with NaCl in SP-Sepharose were dialyzed and subjected to cation exchange chromatography using CM-Sepharose, as described in the Materials and Methods. The protein was eluted with a linear gradient of NaCl (dashed line). The fractions indicated by horizontal bars were separately pooled, concentrated, and subjected to molecular exclusion chromatography using Superdex 75 HiLoad; (**b**) Purification of nigrin-RP1. The fractions marked with NgRP1 in panel **a** were concentrated and subjected to chromatography using Superdex 75 HiLoad. The fractions indicated with the horizontal bar (NgRP1) were pooled, and the traces of nigrin l were eliminated by chromatography using AT-Sepharose, as indicated in the Materials and Methods; (**c**) Purification of nigrin-RP2 and nigrin-RP4. The fractions indicated with NgRP2/4 in panel (**a**) were concentrated and subjected to chromatography using Superdex 75 HiLoad. The fractions indicated with the horizontal bars (NgRP2 and NgRP4) were pooled and the contaminant traces were removed by re-chromatographing the proteins on the same column; (**d**) Purification of nigritin l. The fractions marked with Ngtl on panel (**a**) were concentrated and subjected to chromatography using Superdex 75 HiLoad. The fractions marked with the horizontal bar (Ngtl) were pooled and the contaminant traces were removed by re-chromatographing the proteins in the same column; (**e**,**f**) Purification of nigrin-RP3. The purification of nigrin-RP3 was conducted using the first preparation, with fractions equivalent to those indicated with NgRP3 in panel (**a**), which were concentrated and subjected to chromatography using Superdex 75 HiLoad (**e**). The fractions indicated with NgRP3 in panel **e** were dialyzed and subjected to chromatography using SP-Sepharose (**f**), which was eluted with an NaCl gradient (dashed line), as described in the Materials and Methods.

The fraction eluted with sodium chloride from the SP-Sepharose column was dialyzed and subjected to cation exchange chromatography using CM-Sepharose with a linear gradient of NaCl. As shown in Figure 3a, CM-Sepharose chromatography resolved several protein peaks. The fractions of the peaks were analyzed by electrophoresis in the presence and absence of 2-mercaptoethanol, and their effect on protein synthesis was tested. Further purification of the peaks yielded five proteins that strongly inhibited protein synthesis. Four of these new proteins corresponded to type 2 RIPs, and were named nigrin-Related Proteins 1–4 (nigrin-RPs 1–4). A new type 1 RIP was also found, and we named it nigritin l. For the purification of these new RIPs, the fractions derived via CM-Sepharose chromatography were collected as shown in Figure 3a, and subjected to subsequent chromatographies separately. Thus, nigrin-RP1 was purified by chromatography using Superdex 75 (Figure 3b). The protein obtained contained traces of nigrin l that were eliminated by chromatography using AT-Sepharose, as indicated in the Materials and Methods section. Nigrin-RP2 and nigrin-RP4 were also purified by chromatography using Superdex 75 (Figure 3c) and the trace contaminants were removed by re-chromatographing the proteins in the same column. To purify nigritin l, the fractions indicated in Figure 3a were subjected to chromatography using Superdex 75 (Figure 3d) and the contaminant traces were removed by re-chromatographing the protein on the same column. Finally, nigrin-RP3 was purified by chromatography using Superdex 75 (Figure 3e) followed by chromatography using SP-Sepharose with an NaCl gradient (Figure 3f). The estimated yields of the current preparation were 2.2, 2.6, 0.29, 0.03 and 1.5 mg per 100 g of leaves for nigrin-RPs 1–4 and nigritin l, respectively.

### 2.2. Characterization of RIPs and Lectins from Elderberry Leaves

**Figure 4:**
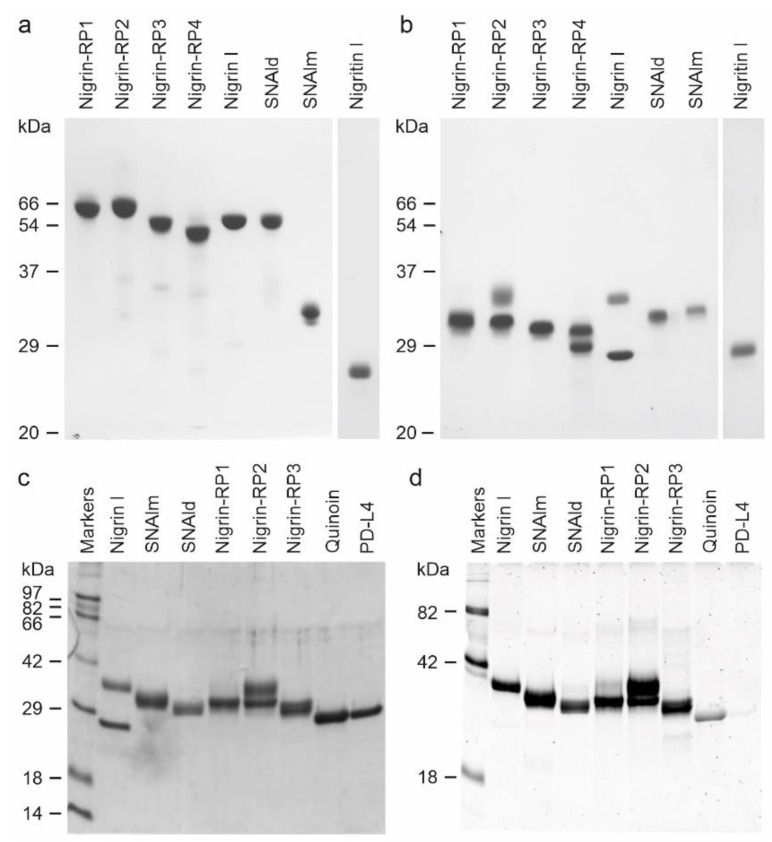
Analysis of purified proteins from elderberry leaves by electrophoresis on polyacrylamide gels: SDS-PAGE of the isolated proteins without (**a**) or with (**b**) 2-mercaptoethanol was carried out on 12% polyacrylamide separating gel and then stained with Coomassie brilliant blue. Samples of 5 µg of each protein were loaded on the gel except for nigritin l, for which 3 µg were loaded. The numbers indicate the corresponding size of the standards in kDa; (**c**,**d**) Sugar staining of RIPs and lectins from *S. nigra* leaves after SDS-PAGE. Each lane contained 5 µg of protein. SDS-PAGE was carried out on a 12% polyacrylamide separating gel in the presence of 2-mercaptoethanol, followed by Coomassie blue staining (**c**), or in-gel glycan detection (**d**) using the Pro-Q Emerald 300 glycoprotein staining kit. Stained glycoproteins were visualized by UV transillumination. Markers: CandyCane™ glycoproteins (**d**) and Coomassie blue (**c**) molecular weight standards.

Purified proteins from elderberry leaves were analyzed by electrophoresis on polyacrylamide gels in the presence of SDS (SDS-PAGE), and in the absence or presence of 2-mercaptoethanol. As shown in Figure 4, in the absence of a reductant, all of the proteins exhibited molecular weights between 50 and 65 kDa except for the lectin, SNAlm, and the type 1 RIP, nigritin l, which exhibited molecular weights of about 32.4 and 25.5 kDa, respectively. In the presence of 2-mercaptoethanol, all of the proteins produced bands with molecular weights between 25 and 35 kDa. Therefore, they are all dimeric proteins except SNAlm and nigritin l, which are monomeric proteins. However, we found that reduction with 2-mercaptoethanol also induced changes in the apparent molecular weight of nigritin l, reducing the mobility of the protein to a molecular weight of about 27.5 kDa, suggesting the presence of intrachain disulfide bonds.

The type 2 RIPs, nigrin l, and nigrin-RPs 1–4 are heterodimeric proteins consisting of a catalytic chain (A chain) and a lectin chain (B chain), both linked through a disulfide bond. In the presence of 2-mercaptoethanol, the apparent molecular weight values obtained for these proteins were 27.3 and 33.7 kDa for the two chains of nigrin l, 30.7 kDa and 33.7 kDa for the two chains of nigrin-RP2, and 27.9 kDa and 29.8 kDa for the two chains of nigrin-RP4. On the other hand, nigrin-RP1 is composed of two subunits of 30.6 kDa and nigrin-RP3 of two subunits of 30.2 kDa. The homodimeric lectin SNAld with an apparent molecular weight of 61.8 kDa contained only a homogeneous protein band of 30.9 kDa in the presence of 2-mercaptoethanol.

Many RIPs and lectins obtained from different species of *Sambucus* are glycoproteins; thus, we studied whether the purified proteins were glycosylated. Figure 4 compares proteins stained with Coomassie blue (Figure 4c) and those detected with a glycoprotein staining kit (Figure 4d). Quinoin (a glycosylated type 1 RIP) and PDL4 (a non-glycosylated type 1 RIP) are shown as controls. It can be observed that all the RIPs and lectins tested are strongly glycosylated, and, in those in which the A chain can be distinguished from the B chain, the latter is the most glycosylated. Thus, while the nigrin l B chain stained for carbohydrate, nigrin l A chain did not show any staining. On the contrary, nigrin-RP2 contained sugar chains on both subunits.

RIPs are potent inhibitors of protein synthesis in eukaryotes, as they are enzymes capable of inactivating ribosomes catalytically. Therefore, in mammalian cell-free systems, they usually show values of IC_50_ (concentration that inhibits 50% protein synthesis) in the ng/mL range. Accordingly, we tested all the proteins isolated from elderberry leaves in a coupled transcription–translation in vitro assay using a rabbit reticulocyte lysate system, finding the following values of IC_50_: 0.36, 0.75, 3.0, 0.35, 0.3 and 6.5 ng/mL for nigrin l, nigrin-RPs 1–4, and nigritin l, respectively (Figure S1). It is worth mentioning that, although all are good inhibitors of protein synthesis, there are great differences among the RIPs displaying IC_50_ values that differ up to 20 times. As expected, the lectins SNAlm and SNAld that lack a catalytic chain did not inhibit protein synthesis up to the maximum tested concentration of 1 μg/mL.

Type 2 RIPs consist of two chains, one being the enzymatic chain and the other being a lectin able to recognize sugars, mostly galactose residues. Due to the lectin activity, type 2 RIPs promote human erythrocyte agglutination. Among proteins isolated from *S. nigra* leaves, only nigrin l and the lectins SNAlm and SNAld can be purified by affinity chromatography using AT-Sepharose 6 B, which exposed galactose residues (Figure 1). The type 2 RIPs, nigrin-RPs 1–4, were not retained on AT-Sepharose (data not shown). We therefore studied the agglutination capacity of nigrin-RPs 1–4 and found that even a concentration of 200 μg/mL did not have any effect on human erythrocytes. Under the same conditions, nigrin l, SNALm, and SNALld agglutinated human erythrocytes at concentrations as low as 12.5, 40, and 6.2 μg /mL, respectively. Therefore, nigrin RPs 1–4, unlike nigrin l, may lack functional sugar binding domains. Similar type 2 RIPs have been previously described in *S. nigra* (SNLRPs 1 and 2) and *S. ebulus* (ebulin-RP) [14,15].

### 2.3. rRNA N-Glycosylase, Adenine Polynucleotide Glycosylase, and DNA Nicking Activities

**Figure 5:**
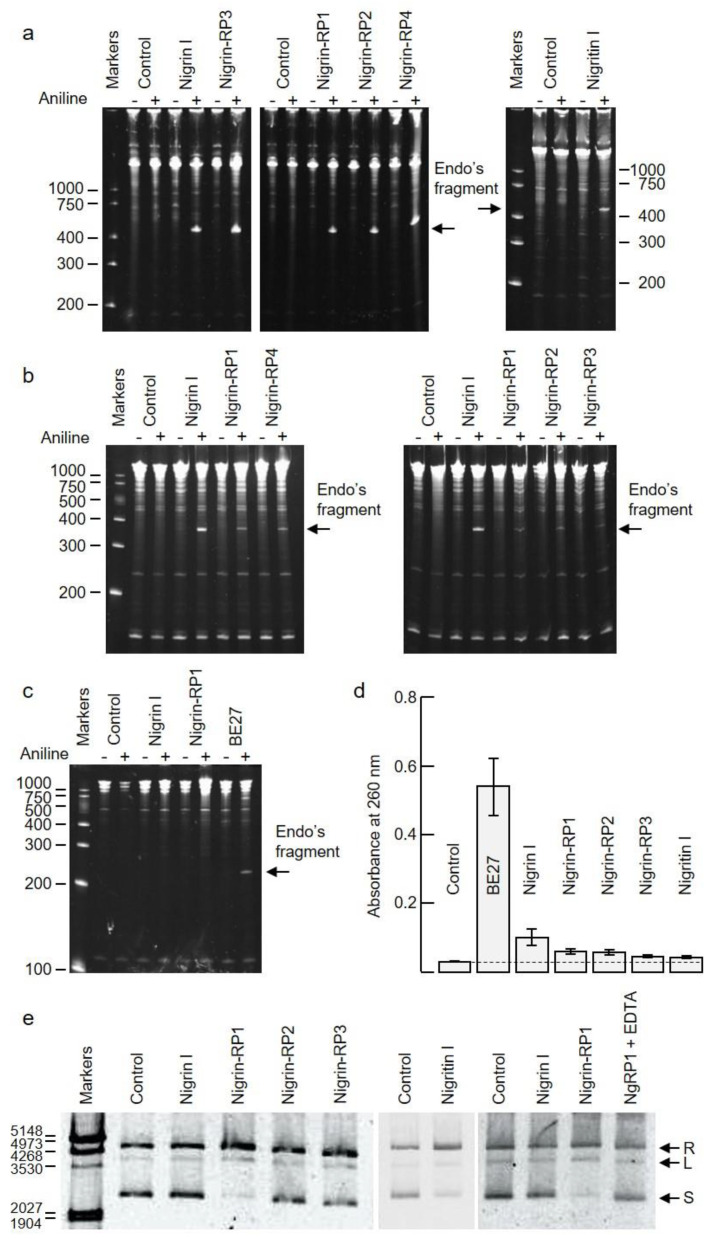
Enzymatic activities of RIPs from *S. nigra* leaves: (**a**–**c**) rRNA N-glycosylase activity in animal, yeast, and bacterial ribosomes. The rRNA-glycosylase activity was tested as indicated in the Materials and Methods. Each lane contained 5 μg of RNA isolated from either untreated (control) or RIP-treated ribosomes from rabbit reticulocyte lysate (**a**), the yeast *Saccharomyces cerevisiae* (**b**), and 1 μg of RNA isolated from the bacterium *Micrococcus lysodeikticus* (**c**). The arrows indicate the RNA fragment (Endo’s fragment) released as a result of the action of RIP after treatment with acid aniline (+). The numbers indicate the size of the markers in nucleotides; (**d**) Adenine polynucleotide glycosylase activity (APG) on DNA from salmon sperm. APG activity of 5 μg of RIP was assayed on salmon sperm DNA as described in the Materials and Methods, and the absorbance of the released adenine was measured at 260 nm. Data represent the mean of two duplicate experiments ± SE; (**e**) Nicking activity on pCR2.1 DNA. Samples comprising 200 ng/10 μL of plasmid DNA were incubated with 5 μg of RIP. Nigrin-RP1 (NgRP1) was also incubated in the presence of EDTA. R, L, and S indicate relaxed, linear, and supercoiled forms of pCR2.1, respectively. The numbers indicate the size of the markers in bp.

RIPs are enzymes that irreversibly inactivate ribosomes because of their N-glycosylase activity (EC 3.2.2.22). The enzyme catalyzes the hydrolysis of the N-glycosidic bond between adenine number 4324 and its ribose in rat ribosomes (or equivalent adenine in sensitive ribosomes of other organisms) [1,2]. This activity can be evidenced by detecting, by means of a polyacrylamide gel electrophoresis, the RNA fragment released (Endo’s fragment or diagnostic fragment) when the apurinic RNA is incubated in the presence of acid aniline [16]. As shown in Figure 5a, the type 2 RIPs, nigrin l and nigrin-RPs 1–4, and the type 1 RIP, nigritin l, cause, after treatment with acid aniline, the release of the diagnostic fragment of 460 nucleotides from rabbit reticulocyte ribosomes. As shown in Figure 5b, the type 2 RIPs can also depurinate yeast ribosomes, releasing a fragment of 368 nucleotides in the presence of acid aniline. Prokaryotic ribosomes are not sensitive to most RIPs. This is the case, for example, for ricin, volkensin [17], and other type 2 RIPs obtained from different species of the genus *Sambucus* [11]. However, they are sensitive to some type 1 RIPs, such as those obtained from *Pytolacca dioica* or *Beta vulgaris* [18,19]. As shown in Figure 5c, BE27 (obtained from the leaves of *B. vulgaris*) releases the diagnostic fragment from ribosomes of *Micrococcus lysodeikticus*, while the type 1 RIP, nigritin l, and the type 2 RIPs (Figure 5c and data not shown, respectively) from elderberry leaves do not, indicating that prokaryote ribosomes are not sensitive to these proteins.

Some RIPs are also capable of removing more than one adenine from rRNA [20], and many of them can depurinate not only rRNA, but also other polynucleotide substrates, such as DNA, poly(A), mRNA, tRNA, and viral RNA. Therefore, the names adenine polynucleotide glycosylase (APG) and polynucleotide–adenosine glycosylase (PNAG) have been proposed for RIPs [21]. RIPs display different APG activities on DNA and RNA, all of which are capable of depurinating DNA from herring and salmon sperm [18,21]. However, this APG activity on DNA varies markedly between different RIPs. Generally, type 2 RIPs, such as ricin and kirkiin, have low activities [22], while some type 1 RIPs, such as those obtained from *Pytolacca dioica* or *Beta vulgaris*, possess very high activities [18,19]. Figure 5d shows the APG activities of RIPs from elderberry leaves on DNA compared with BE27 activity, measured as the absorbance at 260 nm produced by adenines released from salmon sperm DNA. As shown, RIPs from elderberry leaves have low activities, similar to ricin, stenodactylin, and kirkiin, compared to BE27 activity. Of these, the most active is nigrin l, which is twice as active as the others. However, none of the elderberry RIPs showed activity on tobacco mosaic virus RNA (data not shown).

Some RIPs exhibit topoisomerase (nicking) activity on plasmid DNA, transforming supercoiled DNA into the relaxed form; we tested whether elderberry RIPs possessed this activity, and found that only nigrin-RP1 and the type 1 RIP, nigritin l, were able to convert the supercoiled PCR 2.1 DNA forms into the relaxed forms (Figure 5e). Such activity was dependent of Mg^2+^ ions because it was inhibited by EDTA (Figure 5e).

Therefore, elderberry leaves contain mainly a type 1 RIP (nigritin l), which displayed rRNA N-glycosylase activity, a type 2 RIP with N-glycosylase activity and lectin activity (nigrin l), four type 2 RIPs with N-glycosylase activity that fail to agglutinate erythrocytes (nigrin-RPs 1–4), and a monomeric (SNAlm) and a dimeric lectin (SNAld).

### 2.4. Peptide Mapping of RIPs from S. nigra Leaves by High-Resolution MS/MS

**Figure 6:**
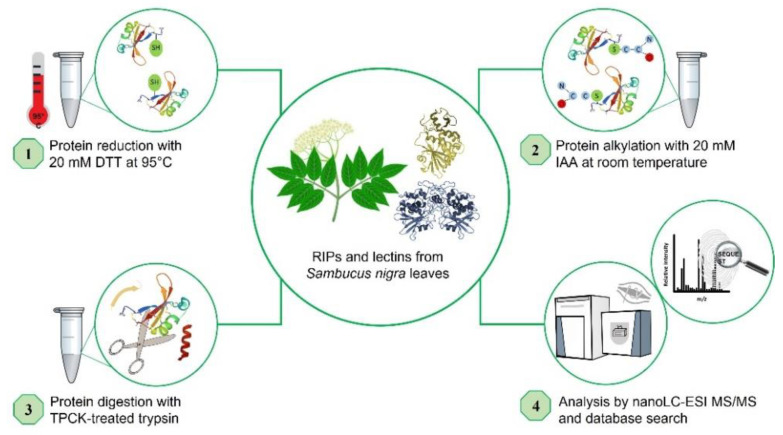
Schematic overview of the experimental workflow used for the peptide mapping of proteins from *Sambucus nigra* leaves by high-resolution nanoLC–MS/MS.

The proteins isolated from *S. nigra* leaves, nigrin l, nigrin-RPs 1–3, SNALm, and SNAld were characterized via a peptide mapping approach based on high-resolution nanoLC–tandem mass spectrometry (Figure 6). Nigrin-RP4 was not studied due to the low levels obtained via the purification process. Similarly, nigritin l was not further investigated due to the lack of sequence data available for database searches. Preliminary optimization of sample preparation steps for MS analyses was performed to define the conditions for the reduction, alkylation, and tryptic hydrolysis of *S. nigra* proteins. An extensive step of protein denaturation and disulfide bridge reduction performed with 20 mM DTT at 95 °C was needed to obtain a significant yield of tryptic peptides suitable for MS analysis. Then, free cysteinyl residue alkylation with iodoacetamide (IAA) and enzymatic proteolysis with trypsin were both performed before nanoLC–ESI–MS/MS analyses on a Q Exactive Orbitrap mass spectrometer. A data-dependent acquisition mode was used, during which higher-energy collisional dissociation (HCD) MS/MS spectra were obtained for the five most intense mass peaks in each scan, allowing for accurate amino acid sequencing of tryptic peptides. By this approach, for the monomeric lectin, 18 peptide spectral matches (PSMs) were mapped on the SNAlm sequence (AC: AAN86132). Amino acid sequences of peptides obtained by high-resolution tandem mass spectrometry are reported in Table S1. Representative MS/MS spectra of peptides are reported in Figure S2. Regarding the dimeric lectin, nine PSMs were mapped on the SNAld sequence (AC: AAN86131, Table S2 and Figure S3).

A high number of MS/MS spectra (53 PSMs) of the type 2 RIP, nigrin l, were mapped on the A chain and B chain of nigrin l (AC: AAN86130, Table S3 and Figure S4).

For both nigrin-RP 1 and 2, sequenced peptides were mapped on SNLRP2 A and B chains (AC: AAC49672, Tables S4 and S5) (23 and 24 PSMs, respectively). On the contrary, nigrin-RP3 was identified as SNLRP1 (AC: AAC49673, Table S6) (27 PSMs). Representative MS/MS spectra of peptides from nigrin-RPs 1, 2, and 3 are reported in Figures S5–S7.

**Figure 7:**
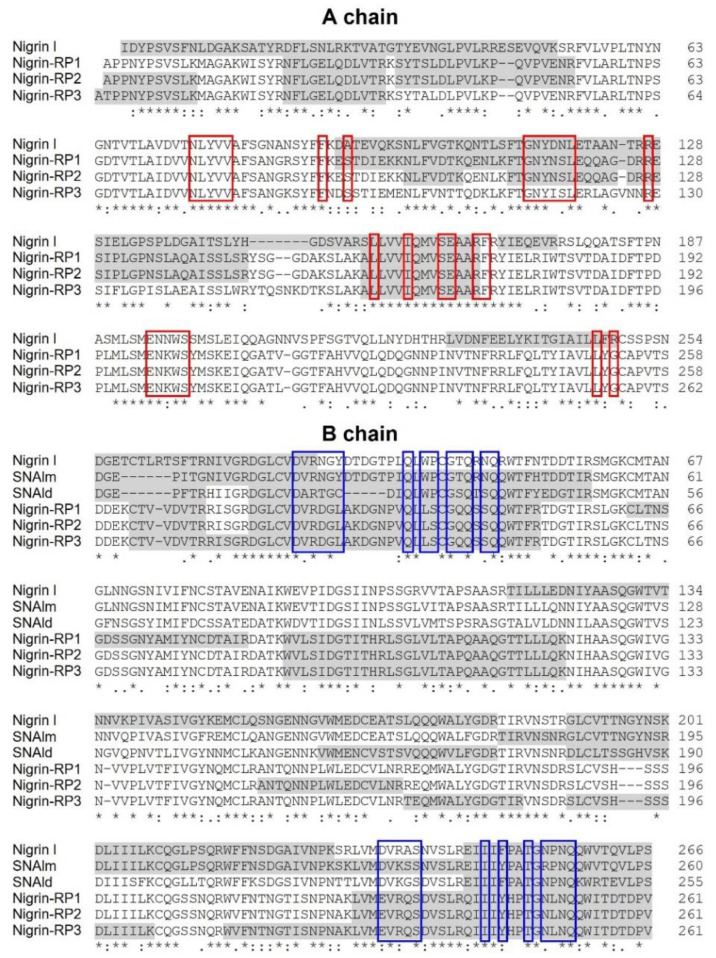
Comparison of the sequences of RIPs and lectins from leaves of *S. nigra* obtained by peptide mapping via high-resolution nanoLC–MS/MS mass spectrometry with sequences deposited in the data banks. The gray shaded sequences were obtained by peptide mapping via high-resolution MS/MS mass spectrometry and match sequences obtained from the data banks with the access numbers AAN86130 (nigrin l), AAN86132 (SNAlm), AAN86131 (SNAld), AAC49672 (nigrin-RP1 and 2), and AAC49673 (nigrin-RP3). The red boxes indicate the amino acids that possibly form the catalytic pocket, and the blue boxes indicate the amino acids that possibly form the 1α and 2γ sugar binding sites. Identical residues (*), conserved substitutions (:), and semiconserved substitutions (.) are reported.

Peptide mapping of proteins purified from *S. nigra* leaves by high-resolution MS/MS identifies the proteins obtained by affinity chromatography using AT-Sepharose with three sequences obtained from *S. nigra* leaves cDNA: the type 2 RIP, nigrin l, and the lectins SNAlm and SNAld (accession numbers AAN86130, AAN86132, and AAN86131, respectively). On the other hand, nigrin-RP3 would be a type 2 RIP homologous to SNLRP1 from elderberry bark (accession number AAC49673), and nigrin-RPs 1 and 2 would be homologous to the type 2 RIP, SNLRP2, also found in the bark of elderberry (accession number AAC49672). Alternatively, nigrin-RPs 1 and 2 could be the same protein with different degrees of glycosylation (see Figure 4d). Evidence against this hypothesis is the fact that these two proteins exhibit different protein synthesis inhibitory activities (IC_50_ fourfold higher for nigrin-RP2) and different behaviors against the supercoiled plasmid (see Figure 5e). Figure 7 graphically presents these data by comparing the sequences obtained by mass spectrometry with the sequences found in the data banks. It is noteworthy that neither SNLRP1 nor SNLRP2 agglutinate erythrocytes [15].

### 2.5. Carbohydrate Binding Properties of Nigrin l, SNAlm, and SNAld

Nigrin l, SNAlm, and SNAld showed hemagglutination activities in human erythrocytes (Section 2.2). To elucidate the sugar binding specificities of these proteins, hemagglutination inhibition with various monosaccharides and disaccharides was carried out (Table 1). The results show that the agglutination produced by the three proteins was inhibited by D-galactose and lactose (β-D-galactopyranosyl-(1→4)-D-glucose). In none of the three proteins was an affinity for D-glucose, D-fructose, D-mannose, or L-fucose observed at the maximum sugar concentration tested. The protein showing the highest affinity for galactose was SNAlm, whereas SNAld showed the lowest affinity. In all cases, lactose inhibited agglutination at a concentration four times lower than galactose.

### 2.6. Structural Analysis of Nigrin l, SNAlm, and SNAld

**Figure 8:**
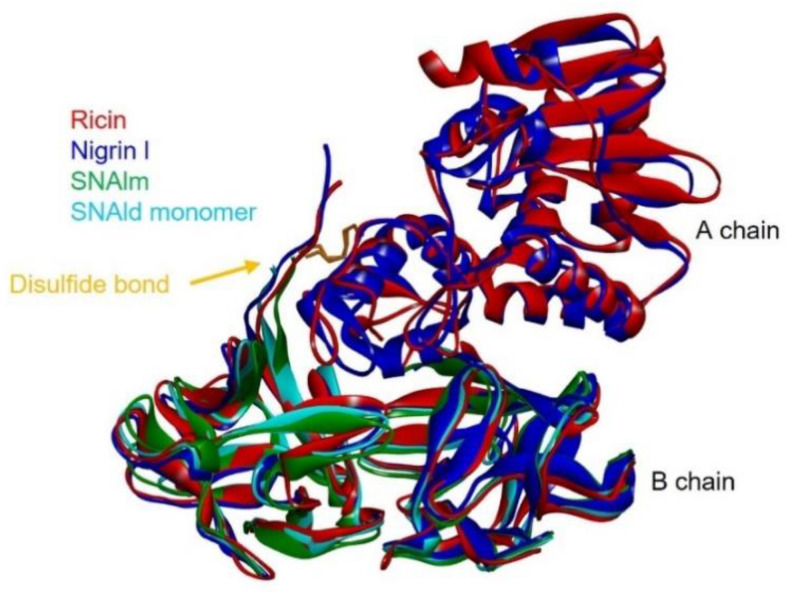
Comparison of the structures of ricin, nigrin l, SNAlm, and the monomer of SNAld. The three-dimensional structural modeling was carried out using AlphaFold2 software, and the figure was generated using Discovery Studio 2021. The arrows indicate the position of the disulfide bond linking A and B chains.

Given the availability of the complete amino acid sequence, it was possible to predict the three-dimensional structures of nigrin l, SNAlm, and SNAld with a computational model using the potentials of deep learning and neural networks [23]. The best three-dimensional models obtained for nigrin l, SNAlm, and SNAld are shown in Figure 8, and all showed local Distance Difference Test (lDTT) values that were much higher than 90%, which makes them suitable for characterizing the binding sites [23]. As described for ricin and other type 2 RIPs [24,25], the A chain of nigrin l consists of three folding domains. The first domain includes the N-terminal, and is composed of six antiparallel β-sheets and two α-helices in the order aAbcdeBf. The second domain consists of five α-helices (helices from C to G). The last domain consists of two α-helices and two antiparallel β-sheets in a α-helix–β-fork–α-helix motif (HghI). Similar to other type 2 RIPs, the B chain is made up of two homologous globular lectin domains arising from gene duplication, which are made up exclusively of β-sheets. Each domain consists of four homologous subdomains (1λ, 1α, 1β, and 1γ for lectin 1; 2λ, 2α, 2β, and 2γ for lectin 2). The subdomains 1λ and 2λ are responsible for the linking to the A chain and the interconnection between the two domains of the B chain, respectively. The subdomains 1α, 1β, and 1γ are arranged in a trefoil structure. This arrangement is also present in lectin 2 with the subdomains 2α, 2β, and 2γ. The 1α and 2γ subdomains contain the two D-galactose binding sites. SNAlm and SNAld have structures similar to nigrin l, but both lack the A chain. In addition, SNAld has an additional cysteine (Cys 23) that allows it to form a dimer with another identical chain.

### 2.7. Molecular Docking

The availability of 3D models of the proteins that allow studies at the molecular level encouraged us to study how D-galactose binds to the 1α and 2γ sites of nigrin l, SNAlm, and SNAld. For this purpose, we carried out a molecular docking study using Autodock 4.2, and compared the results with those already published for ricin [26].

**Figure 9:**
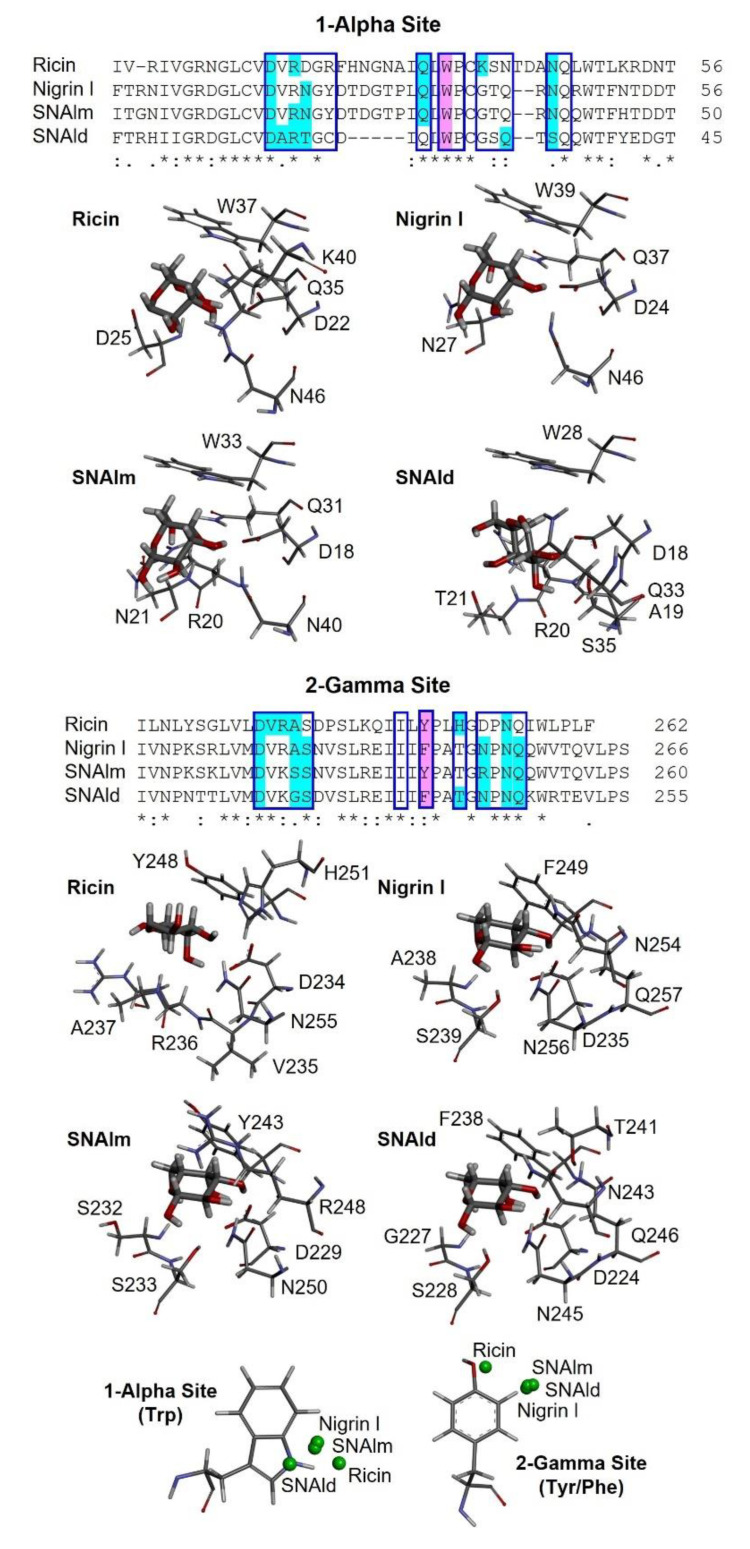
Three-dimensional models of the galactose binding sites of ricin, nigrin l, SNAlm, and SNAld. The alignment of the sequences of subdomains 1α and 2γ of ricin, nigrin l, SNAlm, and SNAld are represented. The binding pockets of sites 1α and 2γ are indicated by blue boxes. Amino acids involved in galactose binding by C–H–π interactions or hydrogen bonds are colored purple and cyan, respectively. Identical residues (*), conserved substitutions (:), and semiconserved substitutions (.) are reported. The galactose binding sites of ricin (PDB 2AAI), nigrin l, SNAlm, and SNAld complexed with β-D-galactopyranose (thick sticks) are represented. The amino acids that bind the galactose molecule, either by C–H–π interactions or both conventional and nonconventional hydrogen bonds, are represented by thin sticks. At the bottom, the positions of the centroids of the pyranosic ring of D-galactose with respect to the aromatic rings of sites 1α and 2γ of ricin, nigrin l, SNAlm, and SNAld are represented by green balls.

As shown in Figure 9, the sequences of the binding sites for sugars of nigrin l, SNAlm, and SNAld are similar to those of ricin. Seven of the fourteen amino acids in the binding pocket of the 1α site are identical, as are seven out of twelve amino acids at the 2γ site. This is consistent with the fact that all these proteins are specific for D-galactose and lactose. However, there are differences with the sugar binding sites of ricin that may influence the affinity of these sites for sugars. The 1α site is identical in nigrin l and SNAlm. In addition, these sites are relatively similar to that of ricin. In all three proteins, the binding of β-D-galactopyranose is the result of the C–H–π interaction between the aromatic rings of tryptophan (W37, W39, and W33 in ricin, nigrin l, and SNAlm, respectively) and the apolar face of the pyranosic ring of galactose. The C–H groups of carbons 3, 4, 5, and 6 are oriented towards the aromatic rings of tryptophan, allowing the π interaction of the electron cloud with the aliphatic protons of sugar that carry a positive partial charge. The polar face of galactose forms hydrogen bonds with five amino acids located on the other side of the binding pocket, four of which (aspartic, arginine, glutamine, and asparagine) are identical in all three proteins. The 1α site of the SNAld is very different. This is likely influenced by the presence within this site of the cysteine (C23) that forms the disulfide bond with the other subunit. Nevertheless, both galactose and lactose can bind at the 1α site of SNAld, although galactose adopts a different arrangement that can affect the affinity for this sugar. In this case, carbons 1, 5, and 6 are oriented towards the aromatic rings of tryptophan, and only coincide, on the other side, the amino acids aspartic and arginine.

At the 2γ site, the amino acids that provide aromatic rings are tyrosine (in ricin and SNAlm) and phenylalanine (in nigrin l and SNAld). However, these residues seem to not affect the orientation of the pyranosic ring at this site, as it is virtually identical. The C–H groups of carbons 4 and 6 are oriented towards the aromatic ring, allowing the interaction with the apolar face of D-galactose, while the polar face forms hydrogen bonds with various amino acids, of which at least four are identical in the three proteins. In ricin, the orientation of galactose is slightly different since the C–H groups of carbons 3, 4, and 5 are oriented towards the aromatic ring, while on the other side, only two amino acids (aspartic and asparagine) coincide.

Similar to D-galactose, lactose can also bind to the 1α and 2γ sites of nigrin l, SNAlm, and SNAld (Figures S8 and S9). However, in all cases, lactose inhibited the agglutination of erythrocytes at a concentration four times lower than D-galactose (Table 1). This is not in accordance with the estimated free energy of binding data provided by Autodock 4.2 (Table S7). One possible explanation is that lactose contains D-galactose in the β-pyranosic form, allowing effective binding to aromatic amino acids at sugar binding sites.

It is worth mentioning that peptide mapping identifies the sequences of the binding sites of nigrin-RPs 1–3 (Figure 7), which match with the sequences of SNLRP1 and SNLRP2 [15]. These are proteins from the bark of *S. nigra* that, similar to nigrin-RPs, do not agglutinate erythrocytes. This was initially attributed to the fact that the 1α and 2γ sites were inactive because amino acid substitutions at these sites prevented carbohydrate binding [15]; however, SNLRP has subsequently been reported to interact with N-acetylglucosamine oligomers, as well as with many glycan structures containing N-acetylglucosamine [27].

### 2.8. Cytotoxic Effect of RIPs from Elderberry Leaves in Cell Cultures

**Figure 10:**
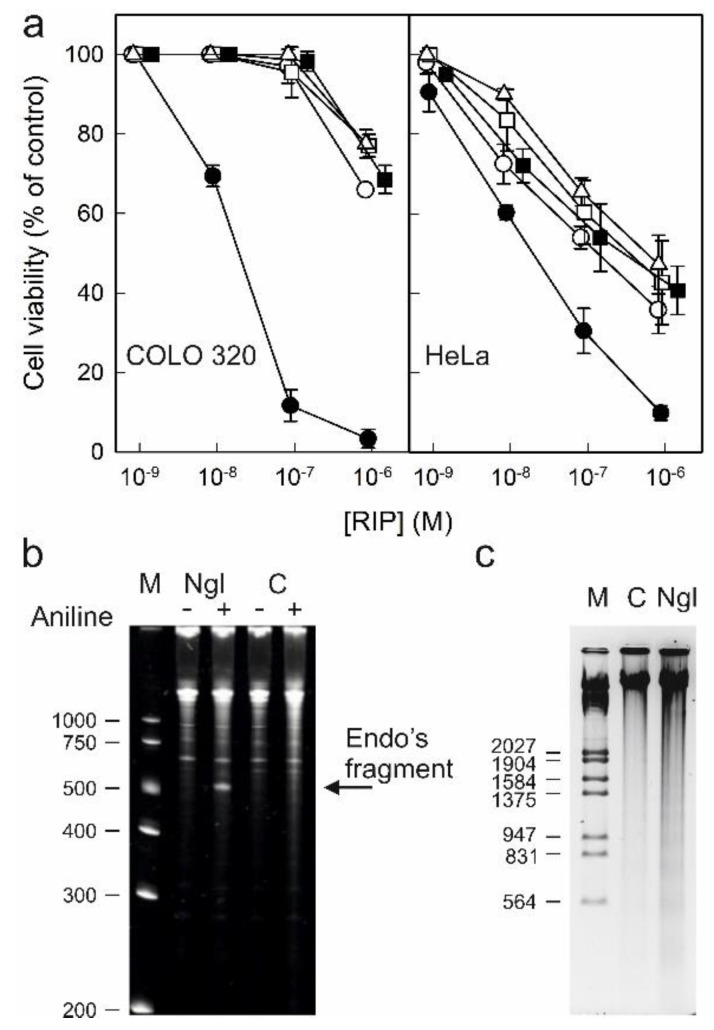
Cytotoxicity of nigrin l, nigrin-RPs 1–3, and nigritin l on HeLa and COLO 320 cells: (**a**) Effect of nigrin l (●), nigrin-RP1 (◯), nigrin-RP2 (□), nigrin-RP3 (△), and nigritin l (■) on viability of COLO 320 (left panel) and HeLa (right panel) cells. Cells were incubated with different concentrations of RIPs for 48 h, and cell viability was evaluated by a colorimetric assay, as indicated in the Materials and Methods. Data represent the mean ± SD of two experiments performed in duplicate; (**b**) N-glycosylase activity of nigrin l on rRNA from HeLa cells. rRNA N-glycosylase activity was assayed as indicated in the Materials and Methods. Each lane contained 2 μg of RNA isolated from either untreated cells (C, control) or cells incubated with 40 nM of nigrin l for 48 h. The arrow indicates the RNA fragment released as a result of RIP action upon acid aniline treatment. Numbers indicate the size of the standards (M) in nucleotides; (**c**) Effect of nigrin l on internucleosomal DNA fragmentation. COLO 320 cells were incubated in the absence (C, control) or presence of 40 nM of nigrin l for 72 h. The DNA was isolated and 4.0 μg was electrophoresed, as indicated in Section 5.3.15. The numbers indicate the corresponding size of the standards (M) (λDNA HindIII/EcoRI) in pb.

Type 1 RIPs consisting of a single enzymatic (A) chain usually display lower toxicity than type 2 RIPs consisting of a binding (B) chain with lectin activity linked to the enzymatic A chain. The carbohydrate binding domains of the B chain recognize glycosylated receptors on the cell surface, facilitating the entry of the A chain into the cell. Ricin is a well-known example of a highly toxic type 2 RIP. However, all type 2 RIPs found in the genus *Sambucus* are considered nontoxic type 2 RIPs since, despite the high enzymatic activity on ribosomes comparable to that of ricin, they show much lower toxicity to cells and animals [3,4,11]. Figure 10a shows the toxicities of RIPs from *S. nigra* leaves towards HeLa and COLO 320 cells. The RIPs from *S. nigra* leaves were toxic to HeLa and COLO 320 cells, exhibiting IC_50_ (concentration of protein causing the death of 50% cells) values ranging from 19 to >1460 nM. In all cases, the cytotoxicity of these RIPs was much less than that exerted by ricin, which affects viability, with IC_50_ values several orders of magnitude lower (0.14–0.6 pM) [14]. The most sensitive were HeLa cells, showing IC_50_ values ranging from 19 to 580 nM, while COLO 320 cells exhibited values between 19 and >1460 nM after 48 h of treatment. When comparing the type 2 RIPs nigrin l, a galactose-binding protein, and nigrin-RPs 1–3, nigrin l was the most active toxin, with IC_50_ values of 19 nM for COLO 320 and HeLa cells. Nigrin-RPs 1–3 displayed very low toxicity, especially on COLO 320 cells, comparable to that of the type 1 RIP, nigritin l. The IC_50_ values obtained from treated HeLa cells were 130, 340, 580, and 280 nM for nigrin-RPs 1–3 and nigritin l, respectively. The lack of sugar binding activity of nigrin-RPs could play a role in their low cytotoxicity. HeLa cells treated with nigrin l, nigrin-RPs, and nigritin l exhibited morphological features characteristic of apoptosis, such as cell rounding and blebbing (data not shown). Several studies reported that the cytotoxicity of RIPs is associated, in addition to rRNA damage, with their ability to induce apoptosis [28]. To investigate the capability of nigrin l to reach the cytosol and inactivate the ribosomes after being endocytosed, we analyzed the ribosomal RNA from HeLa cells treated with the RIP for 48 h. Figure 10b shows that the ribosomes were depurinated, releasing the diagnostic fragment after treatment of the RNA with acid aniline, indicating that nigrin l was able to reach the ribosomes to inhibit protein synthesis. Apoptosis might be a consequence of the ribotoxic stress induced by the RIP after entry into the cytosol, or both processes could run in parallel. To determine whether the observed cytotoxic effects of nigrin l were also mediated via apoptosis in COLO 320 cells, we evaluated the breakdown of the nuclear DNA into oligonucleosomal fragments. As shown in Figure 10c, when COLO cells were treated with 40 nM nigrin l for 72 h, the cleavage of the chromosomal DNA was clearly observed. Thus, our data suggest that the apoptotic pathway was involved in the cell death mediated by RIPs from *S. nigra* leaves.

## 3. Discussion

Species of the genus *Sambucus* are one of the best sources of ribosome-inactivating proteins. From different tissues, type 1 RIPs, type 2 RIPs (heterodimeric and tetrameric), and lectins (monomeric and dimeric) related to the B chain of type 2 RIPs have been obtained [3,11]. Type 2 RIPs isolated from *Sambucus* are peculiar in that they lack the toxicity of other type 2 RIPs, such as ricin [29], abrin [30], stenodactylin [28], and kirkiin [22]. For example, nigrin b is 50,000 times less toxic than ricin to HeLa cells, and 1,500 times less toxic to mice [31]. Therefore, they have been used as the enzymatic component of immunotoxins and other conjugates directed against tumor cells [3,11]. Due to their specificity for the α2,6-linked sialic acid, SNAI from the bark of *S. nigra* and SSA from the bark of *S. sieboldiana* are used in highly diverse biomedical applications, such as diagnosis by ELISA [32,33], histochemistry [34,35], confocal fluorescence microscopy [36], microarrays [37], and new therapeutic strategies [38]. Elderberry RIPs have also been used in agriculture to obtain transgenic plants resistant to viruses [39,40] and insects [41,42].

Most of these proteins have been obtained from *S. nigra*. Thus, from the bark of this species, a galactose-specific type 2 RIP (nigrin b or SNAV) [43,44]; a sialic-acid-specific type 2 RIP (SNAI’) [45]; three type 2 RIPs that do not agglutinate erythrocytes, and that could be specific for N-acetylglucosamine (SNLRP1, SNLRP2, and basic nigrin b) [15,27,46]; a sialic-acid-specific tetrameric type 2 RIP (SNAI) [47]; and a galactose-specific monomeric lectin (SNAII) [48] have been purified. From the fruits two type 1 RIPs (nigritins f1 and f2) [49], a galactose-specific type 2 RIP (nigrin f) [50], a sialic-acid-specific tetrameric type 2 RIP (SNAIf) [51], and a galactose-specific monomeric lectin (SNAIV or SNAIVf) have been obtained [52]. From the seeds, nigrin s (type 2 RIP) [53] and SNAIII (monomeric lectin) [54], both specific for galactose, have been purified. Finally, the presence of SNAflu-I, a tetrameric type 2 RIP reported as being specific for galactose, has been described in the flowers of *S. nigra* [55,56]. Some of them can be considered isoforms; for example, SNAI from the bark and SNAIf from fruits have amino acid identities of 95%, and can therefore be considered as tissue-specific isoenzymes.

However, even though elderberry has been the subject of intense research for the search and study of RIPs and lectins since 1984 [57], leaf proteins have never been purified, despite crude leaf extracts exhibiting very potent activities in both protein synthesis inhibition in rabbit reticulocyte lysate (IC_50_ = 50 ng/mL) and hemagglutination (minimum concentration agglutinating erythrocytes = 1 mg/mL) (data not shown). One of the reasons for this is the difficulty of isolating a considerable number of proteins with similar characteristics, and which also present great developmental variations in their expression in this tissue (data not shown). In spite of this, we proposed to investigate the presence of RIPs and lectins in the leaves in order to isolate and characterize them to enhance our knowledge of these proteins.

We found three proteins that are specific for galactose: nigrin l (a heterodimeric type 2 RIP with an A chain of 27.3 kDa and a B chain of 33.7 kDa), SNAlm (a 32.4 kDa monomeric lectin), and SNAld (a homodimeric lectin with two identical 30.9 kDa subunits) (Figure 4a,b). These proteins could be identified by peptide mapping with three sequences obtained from *S. nigra* leaves cDNA. The sequenced peptides matched with the sequences with access numbers AAN86130 (nigrin l), AAN86132 (SNAlm), and AAN86131 (SNAld), with coverages of 59, 53, and 38%, respectively (Figure 7). Nigrin l can be considered an isoenzyme of nigrin b from the bark since they have an amino acid identity of 98.4%. Likewise, SNAlm can be considered an isoform of SNAIV (or SNAIVf) from fruits, with which it shares 90.2% of the amino acids. However, no dimeric lectins have been found in other elderberry tissues; thus, the most related protein is SELld, found in the leaves of *S. ebulus* [58], with which it presents a homology of 89.9%. Therefore, these dimeric lectins appear to be unique to the leaves. It is also noteworthy that in leaves, no tetrameric type 2 RIPs corresponding to SNAI from the bark, SNAIf from the fruits, or SNAflu-I from flowers were found, tissues in which this type of structure is among the predominant [13,51,55,56,57].

We also found four heterodimeric type 2 RIPs that fail to agglutinate erythrocytes (nigrin-RPs 1, 2, 3, and 4). Nigrin-RPs 1 and 3 are heterodimers whose A and B subunits have a similar molecular weight of about 30 kDa, whereas nigrin-RPs 2 and 4 are heterodimers whose A and B subunits have molecular weights of 30.7 and 33.7 kDa for nigrin-RP2 and 27.9 and 29.8 kDa for nigrin-RP4 (Figure 4a,b). All these proteins are strongly glycosylated, a characteristic they share with most RIPs and lectins from the genus *Sambucus* [14,46,49,50,59,60]. Peptide mapping identified nigrin-RP3 as an isoform of SNLRP1 from the bark, and nigrins-RPs 1 and 2 as SNLRP2 isoforms (Figure 7). Unfortunately, not enough nigrin-RP4 was obtained to perform peptide mapping. The data indicate that nigrin-RPs 1 and 2 could be isoforms with different amino acid sequences or proteins with the same sequence, but different states of glycosylation. In favor of the latter hypothesis is the fact that no differences in the amino acid sequences were found in peptide mapping (Figure 7). Moreover, the B chain of nigrin-RP2 is strongly glycosylated (Figure 4d), suggesting the occurrence of a different glycosylation pattern for the B chain of nigrin-RP2 compared to that of nigrin-RP1 (Figure 4d). This could also explain the different behaviors of the two proteins when interacting with the CM-Sepharose chromatography column (Figure 3a) and in SDS-PAGE (Figure 4). However, the evidence that the two proteins display different enzymatic activities, with nigrin-RP1 being more active, both as an inhibitor of protein synthesis and in nicking activity, leads us to hypothesize that they are probably proteins with different amino acid sequences. Finally, we also found a type 1 RIP, nigritin l, which we have not been able to map. However, based on its behavior in chromatography columns, electrophoresis, and its enzymatic activities (for example, its nicking activity), it could very likely correspond to an isoform of nigritin f1, a protein isolated from fruits of *S. nigra* [49].

One of the most important features of RIPs to consider is their enzymatic properties, both for their possible biological role and for their biotechnological applications. RIPs are inhibitors of protein synthesis using rRNA N-glycosylase activity, which catalyzes the elimination of a specific adenine located in the sarcin–ricin loop (SRL) that is present in the large rRNA of eukaryotes and prokaryotes [1,2]. All the RIPs from elderberry leaves were shown as strong inhibitors of protein synthesis in rabbit reticulocyte lysate, being the most potent nigrin l, nigrin-RP3 and nigrin-RP4 which presented an IC_50_ 20-fold lower than nigritin l. Since the sequence of the active site is very similar in all proteins (Figure 7), the difference could be attributed to small differences in the active site, but mainly to differences in the amino acid sequence of the A chain that binds to ribosomal proteins [61,62]. All RIPs in this study showed rRNA N-glycosylase activity, not only in rabbit reticulocyte ribosomes, but also in yeast ribosomes (Figure 5a,b). However, unlike BE27, none showed activity against bacterial ribosomes (Figure 5c). This is often considered an advantage for biotechnological applications because it facilitates their cloning and expression in bacteria.

Although RIPs are classified as rRNA N-glycosylases, one very important enzymatic activity of these proteins is the depurination of nucleic acids (adenine polynucleotide glycosylase or APG activity) [21]. Different RIPs show different APG activities on DNA and RNA; however, all of them can depurinate the DNA of herring and/or salmon sperm, and some of them can also depurinate various types of RNA [18,19,21]. Although this activity has only been demonstrated in vitro, it could be important for apoptotic activity against animal cells or antiviral activity [19]. All RIPs tested showed APG activity on salmon sperm DNA (Figure 5d), similar to that of ricin and kirkiin [22], although much lower than that of BE27 [19], since type 1 RIPs usually have an APG activity on DNA 10-fold higher than type 2 RIPs [21]. Of all the RIPs in elderberry leaves, the one that displayed the highest activity was nigrin l, which showed a twofold higher activity with respect to the other proteins.

Some RIPs show endonuclease (nicking) activity on the DNA of supercoiled plasmids that produces relaxed and sometimes linear plasmids. This ability may be necessary for these proteins to perform different biological functions, including resistance to pathogenic microorganisms or viruses [18,19]. Only nigrin-RP1 and nigritin l promoted the conversion of supercoiled DNA into relaxed forms. This activity was dependent on magnesium ions, as it was inhibited by EDTA, according to what has been reported for other RIPs [18,19]. Regarding this activity, nigrin-RP1 and nigrin-RP2 showed very different behaviors, so the differences between both proteins must be more than just a difference in the glycosylation pattern. Many RIPs do not present this activity, which is related to a different configuration in the structure near the active site that allows the accommodation of the supercoiled DNA of the plasmids [19,63].

Nigrin l, SNAlm, and SNAld were revealed to be lectins with affinities for galactose (Table 1), the affinity of SNAlm for both galactose and lactose being significantly higher. This contrasts with some elderberry RIPs that have affinities for sialic acid, and appear not to be present in elderberry leaves. Due to the arrangement of its hydroxyl groups, β-D-galactopyranose has two faces, a polar face and an apolar face. In several RIPs such as ricin, abrin, stenodactylin, and kirkiin, the binding of β-D-galactopyranose to the 1α and 2γ sites is the result of the C–H–π interaction between the aromatic ring(s) of an amino acid (tryptophan, phenylalanine, tyrosine, or histidine) and the apolar face of the pyranosic ring of galactose, and hydrogen bonds between the hydroxyl groups of the polar face and amino acids located on the other side of the pocket of binding [24,25].

The recent emergence of programs that predict the structure of proteins at the atomic level [23,64] has allowed us to conduct in silico experiments to predict how galactose and lactose bind to these proteins. A surprising finding is that the 1α site of the SNAld is fully functional, although the subdomain in which it is located is also used for the linking of the two monomers that form the dimeric protein (Figure 9). In SNAld, the 1α site has undergone numerous changes, including the deletion of five amino acids, which allows it to keep away the 1α site of the other subunit without affecting the galactose binding capacity (Figure S10). The way galactose binds to the 1α site of nigrin l and SNAlm is practically identical, and very similar to that of ricin, although some of the amino acids with which it forms hydrogen bonds change, which could affect the affinity for sugars (Figure 9). However, the orientation of galactose at the 1α site of SNAld is different, caused by what we have discussed above, which does not seem to affect the affinity for monosaccharide with respect to the other two proteins. In the case of the 2γ site, the way galactose binds to the three elderberry proteins is practically identical, and somewhat different from that of ricin (Figure 9). It has been suggested that the difference between toxic RIPs, such as ricin, and nontoxic RIPs such as ebulin l or nigrin b, lies in the 2γ site, which binds galactose in a different way, resulting in a decreased affinity for membrane glycoproteins containing galactose and, as a consequence of the deficient binding, an intracellular pathway different from that of ricin [3,11]. The most notable change at this site is the substitution of tyrosine for phenylalanine, which seemed to suggest that this change alone could modify the way galactose binds. In the case of ebulin l, this prevents the binding of lactose, which explains the fact that this protein has less affinity for the α-lactose-agarose gel [65]. Nigrin b, which has a 98.4% homology with nigrin l, also has less affinity for the AT-Sepharose matrix [13]; however, we have not observed that the binding of galactose to the 2γ site of nigrin l is affected by glucose as part of lactose (Figure S9). Nor does the change from tyrosine to phenylalanine seem to be decisive because SNAlm contains tyrosine and does not exhibit a different mode of galactose binding from that of nigrin l. However, this different affinity could be explained by the different way galactose binds to this site with respect to ricin (Figure 9).

Peptide mapping identified the sequence of the binding sites of nigrin-RPs l, 2, and 3 with the sequences of SNLRP1 and SNLRP2, type 2 RIPs that fail to agglutinate erythrocytes and do not recognize galactose [15]. In these proteins, the most prominent change occurs at the 1α site, which lacks an aromatic amino acid. The 2γ site contains tyrosine; however, several changes occur in the amino acids that form hydrogen bonds with galactose, which could affect the monosaccharide binding to this site. On the other hand, ebulin-RP is a heterodimeric type 2 RIP present in *S. ebulus* leaves that displays rRNA N-glycosylase activity but lacks functional sugar binding domains [14]. Moreover, the B chain of ebulin-RP shares a strong homology (83.14%) with the B chains of SNLRPs, and thus with those of nigrin-RPs. It has been proposed, based on molecular docking, that the loss of lectin activity of ebulin-RP may be due to the presence of inactive 1α and 2γ sites, and this could also be the case for nigrin-RPs from leaves.

We have performed a study of the cytotoxic activity of *S. nigra* leaf RIPs, including the type 2 RIPs (nigrin l and nigrin-RPs 1–3) and the type 1 nigritin l, towards COLO 320 and HeLa cells. First, our data confirmed that the elderberry type 2 RIPs are much less toxic than ricin, which affects the viability of these cells, with IC_50_ five–six orders of magnitude lower [14]. The reason for the different toxicities among type 2 RIPs is not clear. Type 2 RIPs from *Sambucus* leaves, inactivate ribosomes in vitro with higher efficiency than ricin, therefore, the different toxicities could be better attributed to differences between the B chains, which are responsible for the interaction with cellular membranes, than to the enzymatic A chains. In this line, it has been shown that the type 2 RIPs from *Sambucus*, ebulin l and nigrin b, bind to cells to a lesser extent than ricin [4,31]. Furthermore, these RIPs have lower affinities for galactose than ricin [4,13,65]. The differential affinity of RIPs from *Sambucus* for galactosides on cell surfaces may determine its intracellular fate and possibly its cytotoxicity. Thus, it has been reported that, unlike ricin, type 2 RIPs from *Sambucus* follow a weakly productive Golgi-independent pathway to the cytosol to intoxicate the cells [4,14,31]. An important observation when comparing the toxicity of nigrin l, nigrin-RPs, and nigritin l towards COLO 320 and HeLa cells is that nigrin l was the most active toxin. Nigrin l is a galactose binding lectin, whereas nigrin-RPs 1–3 failed to bind to the affinity matrix of AT-Sepharose and to agglutinate erythrocytes. Therefore, the low cytotoxicity of nigrin-RPs compared to nigrin l could be related to deficient sugar binding domains, which is the major difference with nigrin l. According to this, the toxicity of nigrin-RPs is comparable to that of the type 1 RIP nigritin l that lacks a B chain. Several studies underline that the cytotoxicity of RIPs is associated with their ability to induce apoptosis [28]. We also found that RIPs from *S. nigra* leaves induced apoptosis. HeLa cells treated with the RIPs showed apoptotic morphological features and, in COLO 320 cells, nigrin l treatment led to oligonucleosomal DNA fragmentation. One of the unresolved questions is whether rRNA damage leading to the inhibition of protein synthesis is solely responsible for this RIP-induced apoptosis. On the one hand, the ribosome may not be the only substrate of RIP action and, on the other hand, in the case of type 2 RIPs, apoptosis might also be induced by lectin binding to specific glycosylated proteins on the cell membrane, leading to activation of cell death factor receptors.

## 4. Conclusions

This work contributes to expanding our knowledge of the family of RIPs and RIP-related lectins produced by *S. nigra*, with eight new proteins found in the leaves. Our results demonstrate the presence in this tissue of a type 2 RIP and two related lectins specific for galactose, four type 2 RIPs with deficient sugar binding domains, and one type 1 RIP.

Although initially only SNAI has been used, with the discovery and study of other proteins of the genus *Sambucus*, its use has been extended to other proteins, such as SSA, ebulin l, nigrin b, and SNAI’. The study of RIPs and related lectins of elderberry leaves completes the knowledge of this type of proteins in this species, and opens new perspectives, not only in the study of the biological functions attributed to them, but also in their use in biomedicine and agriculture.

New techniques such as protein structure prediction based on deep learning and neural networks, and peptide mapping based on high-resolution nanoLC–tandem mass spectrometry, can be very useful tools to further advance our knowledge of these types of proteins.

## 5. Materials and Methods

### 5.1. Materials

The sources of the chemicals were described previously [16]. Leaves from elder were harvested at Cobos de Cerrato (Palencia, Spain) in early summer. CM-Sepharose FF, Q-Sepharose FF, CM-Sepharose FF, Sepharose 6 B, and Superdex−75 HiLoad 26/60 columns were purchased from GE Healthcare (Barcelona, Spain). The acid-treated Sepharose (AT-Sepharose) was prepared as described in [66], treating the Sepharose 6 B with 0.1 N HCl at 50 °C for 3 h. The gel was then washed with water (Elix 5, Millipore) until a neutral pH was obtained, and stored in water at 4 °C until it was used. Tosyl phenylalanyl chloromethyl ketone (TPCK)-treated trypsin was purchased from Merk Life Science S.r.l. (Milan, Italy). Acetonitrile (CH3CN), formic acid (FA), and water (LC–MS grade) were from Fisher Scientific Italia (Milan, Italy). Century™-Plus RNA Markers were purchased from Fisher Scientific (Madrid, Spain). Rabbit reticulocyte lysate system (nuclease-treated) was purchased from Promega Biotech Iberica S.L. (Alcobendas, Madrid, Spain).

### 5.2. Cell Lines and Culture

COLO 320 (human colon carcinoma) and HeLa (human cervix epithelioid carcinoma) cells were obtained from the European Culture Collection (ECACC) and grown in RPMI 1640 medium (GIBCO BRL, Barcelona, Spain) supplemented with 10% fetal bovine serum (FBS), 100 U/mL penicillin, and 0.1 mg/mL streptomycin, under 5% CO_2_ at 37 °C.

### 5.3. Methods

#### 5.3.1. Preparation of Crude and Acidified Extracts and Chromatography Using SP-Sepharose

Two extracts were prepared from 700 and 540 g of *S. nigra* leaves. The leaves were crushed with dry ice in a crusher (Sammic Cutter K−52) and extracted with eight volumes of PBS (5 mM sodium phosphate, pH 7.5, 0.14 M NaCl) overnight at 4 °C. The crude extract was clarified by filtering it through a nylon mesh and then centrifuging it for 30 min at 9000 rpm in a JA−10 rotor (12,900× *g*) at 2 °C. The crude extract was acidified by adding glacial acetic acid to a pH of 4.05, and clarified again by filtering it through a nylon mesh and centrifuging it under the same conditions. The acidified crude extract was loaded onto a SP-Sepharose FF column (25 × 5 cm = 491 mL) equilibrated with 10 mM sodium acetate (pH 4.5). The column was washed at a flow rate of 7 mL/min with the same buffer until the absorbance at 280 nm of eluent dropped to almost zero. Proteins were first eluted with 5 mM sodium phosphate (pH 6.66) and then with the same buffer containing 1 M NaCl. Protein elution was controlled by measuring the absorbance at 280 nm, and the fractions eluted with sodium phosphate and NaCl were collected separately.

#### 5.3.2. Purification of Nigrin l, SNAlm, and SNAld

The protein eluted with sodium phosphate from the first preparation (1116 mg) was supplemented with 0.28 M NaCl and subjected to chromatography using AT-Sepharose (25 × 5 cm = 137 mL) equilibrated with 5 mM sodium phosphate (pH 7.5) containing 0.28 M NaCl. The column was kept at 0 °C and three identical chromatographies were performed, each loading 372 mg of protein. The column was washed at a flow rate of 4.5 mL/min with sodium phosphate 5 mM (pH 7.5) containing 0.28 M NaCl until the absorbance at 280 nm dropped to almost zero, and was eluted with the same buffer containing 0.2 M lactose. The eluate from the three chromatographies (218 mg of protein) was combined and concentrated up to 17 mL using an Amicon YM10 membrane. Next, three aliquots were prepared, each of which was subjected to molecular exclusion chromatography using a Superdex 75 HiLoad 26/60 (60 × 2.6 cm = 319 mL) column equilibrated with PBS at a flow rate of 2 mL/min. Fractions of 5 mL were collected, and their composition was determined by SDS-PAGE in the presence and absence of 2-mercaptoethanol. Fractions containing SNAlm were collected, dialyzed against water, frozen, and freeze dried; 84 mg of lyophilized protein was obtained. Fractions containing nigrin l and SNAld were collected, concentrated, and re-chromatographed on the same column to remove traces of SNAlm, dialyzed. Subsequently, these fractions, in 5 mM sodium phosphate (pH 7.5), were subjected to anion exchange chromatography using Q-Sepharose FF (5 × 1.6 cm = 10 mL) equilibrated with 5 mM sodium phosphate (pH 7.5) at a flow rate of 4 mL/min. After loading the sample and washing with sodium phosphate buffer, the protein was eluted with a linear gradient of 600 mL of NaCl from 0 to 0.6 M. Fractions of 8 mL were collected, and the fractions containing nigrin l were pooled together, dialyzed against water, frozen, and freeze dried. The SNAld was further purified by repeating the same chromatography procedure; 14 mg of nigrin l and 10 mg of SNAld were obtained.

#### 5.3.3. Purification of Nigrin-RPs 1, 2, 3, and 4, and Nigritin l

The protein eluted with NaCl from the second preparation (1820 mg) was dialyzed and subjected to cation exchange chromatography using CM-Sepharose FF (7 × 2.6 cm = 37 mL) equilibrated with 5 mM sodium phosphate (pH 6.66) at a flow rate of 7 mL/min. After loading the sample and washing with sodium phosphate, the protein was eluted with a linear gradient of 1596 mL of NaCl from 0 to 0.3 M. Fractions of 10.5 mL were collected, which were tested via protein synthesis, and analyzed by SDS-PAGE. Fractions containing nigrin-RPs 1–4 and nigritin l were placed together, concentrated using an Amicon YM10 membrane, and separately subjected to molecular exclusion chromatographies through a Superdex 75 HiLoad 26/60 column equilibrated with PBS at a flow rate of 2 mL/min. Fractions of 5 mL were collected, and their composition was determined by SDS-PAGE in the presence and absence of 2-mercaptoethanol. Nigrin-RP1 was subjected to chromatography using AT-Sepharose (5 × 4 cm = 20 mL) to remove traces of nigrin l; the fraction not retained by the AT-Sepharose was dialyzed against water and freeze dried, obtaining 12 mg of lyophilized protein. Both the fractions containing nigrin-RP2 and those containing nigrin-RP4 were re-chromatographed using a Superdex 75 HiLoad column to eliminate traces contaminating the other protein, obtaining 14 and 0.15 mg of nigrin-RP2 and nigrin-RP4, respectively. The purification of nigrin-RP3 was conducted using the first preparation. After molecular exclusion chromatography, the protein was dialyzed and purified to homogeneity by chromatography using SP-Sepharose FF (4.5 × 1 cm = 3.5 mL) equilibrated with 5 mM sodium phosphate (pH 7.5) at a flow rate of 1 mL/min. After loading the sample and washing with sodium phosphate, the protein was eluted with a linear gradient of 140 mL of NaCl from 0 to 0.2 M. Fractions of 2 mL were collected and the fractions containing nigrin-RP3 were placed together, dialyzed against water, frozen, and freeze dried, obtaining 2 mg of lyophilized protein. Nigritin l was re-chromatographed using a Superdex 75 HiLoad column to remove traces of contaminants, dialyzed, and freeze dried, obtaining 8 mg of lyophilized protein.

#### 5.3.4. Analytical Procedures

Protein concentrations were determined using the spectrophotometric method of Kalb and Bernlohr [67]. Analyses of proteins by SDS-PAGE were carried out as described elsewhere [68] using 12% acrylamide gels and the Hoefer™ MiniVE system (Thermo Fisher Scientific-ES, Madrid, Spain). The glycosylation analysis was performed in gel after SDS-PAGE with a Mini-Protean II system (Bio-Rad; Milan, Italy) using the Pro-Q emerald 300 Glycoprot Probes Kombo (Life Technologies, Monza, Italy). Glycosylated proteins were visualized using the ChemiDoc™ XRS system.

#### 5.3.5. Assays of Cell-Free Protein Synthesis

The effect of RIPs on protein synthesis was determined through a coupled transcription–translation in vitro assay using a rabbit reticulocyte lysate system [13]. The reaction mixture contained 0.6 μL of rabbit reticulocyte lysate and 5.8 μL of a mixture of the following components: 4.6 U ribonuclease inhibitor, 2.3 U T7 RNA polymerase, 0.2 μg luciferase T7 plasmid, rNTPs (0.4 mM each), amino acids (2 μM each), 10 mM Tris–HCl (pH 7.8), 0.2 mM spermidine, 28 mM KCl, 1 mM MgCl_2_, and nuclease-free water. The mixtures were incubated at 30 °C for 10 min and placed on ice. Then, 1.6 μL of either water or different protein concentrations were added and the sample mixture was incubated at 30 °C for 40 min. Subsequently, 25 μL water was added and mixed with 28 μL of Luciferase Assay Reagent (Promega, Alcobendas, Madrid, Spain) at room temperature. Luminescence was determined with a Junior LB 9509 luminometer (Berthold Technologies GmbH & Co. KG, Bad Wildbad, Germany). Three experiments were conducted in duplicate, and IC_50_ (concentration that inhibits 50% protein synthesis) values were calculated by linear regression.

#### 5.3.6. rRNA N-Glycosylase Assays on Rabbit Reticulocyte, Yeast, Bacterium Lysates, and HeLa Cells

rRNA N-glycosylase assays were conducted as described elsewhere [16]. Rabbit reticulocyte lysate (40 μL) was incubated with 5 μg of RIP at 30 °C for 1 h. N-glycosylase activity on *Saccharomyces cerevisiae* ribosomes was assayed in 50 μL samples of S−30 lysate from yeast in 10 mM Tris–HCl buffer (pH 7.6) containing 10 mM KCl, 10 mM magnesium acetate, and 6 mM 2-mercaptoethanol, which was incubated with 5 μg of RIPs at 30 °C for 1 h. N-glycosylase activity on *Micrococcus lysodeikticus* ribosomes was assayed using 100 μL of bacterial lysate samples in 20 mM Tris–HCl buffer (pH 7.8), which were incubated with 5 μg of RIP at 30 °C for 1 h. After treatment, the RNA was extracted by phenolization, treated with 1 M aniline acetate (pH 4.5), and precipitated with ethanol. HeLa cells (1 × 10^6^/plate) were incubated for 48 h in the presence of 40 nM of nigrin l. After treatment, cells were harvested by centrifugation at 1000× *g* for 5 min. The pellets were lysed, and the RNA was isolated following the instruction of the RNeasy Mini Kit (Qiagen GmbH, Hilden, Germany). RNA was treated with 1 M aniline acetate (pH 4.5) for 10 min at 0 °C and precipitated with ethanol. The RNAs were subjected to electrophoresis at 15 mA for 2 h (rabbit and HeLa cells) or 1 h 30 min (yeast and bacterium) in a 7 M urea/5% (*w*/*v*) polyacrylamide gel and stained with GelRed nucleic acid stain (Biotium Inc., Hayward, CA, USA) [16].

#### 5.3.7. Adenine Polynucleotide Glycosylase Activity on Salmon Sperm DNA and Tobacco Mosaic Virus (TMV) RNA

The adenine release was measured according to the method reported elsewhere [69] with a few modifications. First, 10 μg of salmon sperm DNA was incubated with 5 μg of RIP in 300 μL of a reaction mixture containing 100 mM KCl and 50 mM magnesium acetate (pH 4), at 30 °C for 2 h. After incubation, the DNA was precipitated with ethanol at −80 °C for 3 h and centrifugated at 13,000 rpm for 15 min. Adenine released from RIP-treated DNA was determined in the supernatants spectrophotometrically at 260 nm. Analysis of the adenine polynucleotide glycosylase activity on tobacco mosaic virus (TMV) RNA was carried out as described elsewhere [18].

#### 5.3.8. DNA Cleavage Experiments

Nicking activity experiments were performed as previously reported [18]. Each reaction contained 5 μg of RIP and 200 ng of pCR2.1 DNA in a final volume of 10 μL, comprised of 10 mM Tris–HCl, 5 mM MgCl_2_, 50 mM NaCl, and 50 mM KCl, pH 7.8. Samples were incubated for 2 h at 30 °C, run on agarose gel (0.8%) in TAE buffer (0.04 M Tris, 0.04 M acetate, 1 mM EDTA, pH 8.0) and visualized by GelRed nucleic acid staining (Biotium Inc., Hayward, CA, USA).

#### 5.3.9. Tryptic Digestion and Sample Preparation for MS/MS Analyses

Aliquots of protein samples (50 µg) were reduced with 20 mM dithiothreitol (DTT, 5 min at 95  °C) and alkylated with 20 mM iodoacetamide (IAA, 30 min, in the dark, at room temperature). Enzymatic hydrolyses were performed on reduced and alkylated samples by adding TPCK-treated trypsin with an enzyme/substrate (E/S) ratio of 1:200 (*w*/*w*) for 3 h, 1:100 for 16 h, and 1:50 for 4 h at 37  °C.

#### 5.3.10. High-Resolution NanoLC–Tandem Mass Spectrometry

Mass spectrometry analyses on tryptic samples (500 fmol) were performed on a Q Exactive Orbitrap mass spectrometer equipped with an EASY-Spray nano-electrospray ion source (Thermo Fisher Scientific, Bremen, Germany) and coupled with a Thermo Scientific Dionex UltiMate 3000 RSLCnano system (Thermo Fisher Scientific). Solvent composition was 0.1% formic acid in water (solvent A) and 0.1% formic acid in acetonitrile (solvent B). Peptides were loaded on a trapping PepMap™100 μCartridge Column C18 (300 μm × 0.5 cm, 5 μm, 100 angstroms) and desalted with solvent A for 3 min at a flow rate of 10 μL/min. After trapping, eluted peptides were separated on an EASY-Spray analytical column (50 cm × 75 μm ID PepMap RSLC C18, 3 μm, 100 angstroms) and heated to 35 °C at a flow rate of 300 nL/min using the following gradient: 4% B for 3 min, from 4% to 55% B in 60 min, from 55% to 70% B in 10 min, and from 70% to 95% B in 2 min. Eluting peptides were analyzed on the Q-Exactive mass spectrometer operating in positive polarity mode with capillary temperature of 280 °C and a potential of 1.9 kV applied to the capillary probe. Full MS survey scan resolution was set to 70,000 with an automatic gain control (AGC) target value of 3 × 10^6^ for a scan range of 375–1500 *m/z* and maximum ion injection time (IT) of 100 ms. The mass (*m*/*z*) 445.12003 was used as lock mass. A data-dependent top 5 method was operated, during which higher-energy collisional dissociation (HCD) spectra were obtained at 17,500 MS2 resolution with AGC target of 1 × 10^5^ for a scan range of 200–2000 *m*/*z*, maximum IT of 55 ms, 2 *m*/*z* isolation width, and normalized collisional energy (NCE) of 27. Precursor ions targeted for HCD were dynamically excluded for 15 s. Full scans and Orbitrap MS/MS scans were acquired in profile mode, whereas ion trap mass spectra were acquired in centroid mode. Charge state recognition was enabled by excluding unassigned charge states.

#### 5.3.11. Data Processing

The acquired raw files were analyzed with Proteome Discoverer 2.4 software (Thermo Fisher Scientific, Rockford, IL, USA) using the SEQUEST HT search engine. The HCD MS/MS spectra were searched against the whole UniProt_SwissProt KB database and against a homemade database including *S. nigra* RIP sequences deposited into the NCBI_GeneBank_NIH assuming trypsin (Full) as digestion enzyme with two allowed numbers of missed cleavage sites. The mass tolerances were set to 10 ppm and 0.02 Da for precursor and fragment ions, respectively. Oxidation of methionine (+15.995 Da) was set as dynamic modification, and carbamidomethylation of cysteine (+57.021 Da) as static modification. False discovery rates (FDRs) for peptide spectral matches (PSMs) were calculated and filtered using the Target Decoy PSM Validator Node in Proteome Discoverer. The Target Decoy PSM Validator Node specifies the PSM confidences based on dynamic score-based thresholds. It calculates the node-dependent score thresholds needed to determine the FDRs, which are provided as input parameters of the node. The Target Decoy PSM Validator was run with the following settings: maximum Delta Cn of 0.05, a strict target FDR of 0.01, a relaxed target FDR of 0.05, and validation based on q-value. The Protein FDR Validator Node in Proteome Discoverer was used to classify protein identifications based on q-value. Proteins with a q-value of <0.01 were classified as high-confidence identifications and proteins with a q-value of 0.01–0.05 were classified as medium-confidence identifications. Only proteins identified with medium or high confidence were retained, resulting in an overall FDR of 5%. All MS/MS spectra were manually validated to assign the best PSM to peptide sequences. When multiple PSM were mapped on the same peptide sequence, those with the highest accuracy were selected.

#### 5.3.12. Hemagglutination Activity and Carbohydrate Binding Properties

Hemagglutination activity (HA) was assayed as described elsewhere [25]. The HA was determined in microtiter plates. Each well contained 50 µL of serial dilutions of the proteins and 50 µL of 1% erythrocyte suspension, and the plates were incubated for 1 h at room temperature. The minimum concentration of protein causing complete agglutination was visually evaluated. For hemagglutination inhibition assay, six sugars (D-glucose, D-galactose, D-fructose, D-mannose, L-fucose, and lactose) were tested for their ability to inhibit the HA of the proteins. Each well contained 50 µL of carbohydrates serially diluted, and 25 µL of the proteins at a concentration one titer higher than the HA dose. An equal volume of 2% erythrocyte suspension (25 µL) was added to each well and incubated for 1 h at room temperature. The minimum concentration of the tested sugars that completely inhibited HA activity was determined.

#### 5.3.13. Protein Structure Studies and Graphical Representation

The structure of ricin (accession number 2 AAI) is available in the Protein Data Bank (https://www.rcsb.org/ (accessed on 26 April 2022). The three-dimensional structural modeling of nigrin l, SNAlm, and SNAld was carried out with the AlphaFold2 software following the instructions of the website https://colab.research.google.com/github/sokrypton/ColabFold/blob/main/AlphaFold2.ipynb#scrollTo=G4yBrceuFbf3 (accessed on 26 May 2022) [23]. The study representations and graphs of protein structures were constructed with the help of the Discovery Studio Visualizer suite (v21.1.0) (https://www.3dsbiovia.com/ (accessed on 26 April 2022). The SNAld dimer model was carried out on the SymmDock server (http://bioinfo3d.cs.tau.ac.il/SymmDock/ (accessed on 19 June 2022)) [70].

#### 5.3.14. Molecular Docking

The structures of β-D-galactose (PubChem CID 439353) and β-lactose (PubChem CID 6134) are available in the PubChem database (https://pubchem.ncbi.nlm.nih.gov/ (accessed on 10 June 2022)) [71]. Docking was carried out using Autodock 4.2 (http://autodock.scripps.edu/ (accessed on 15 October 2021)) [72], as described elsewhere [25]. The docking of D-galactose was performed on a grid of 120 × 120 × 120 points, with the addition of a central grid point. The grid was centered on the centroid of the pyranosic ring of galactose at the 1α or 2γ sites of the ricin structure (accession number 2AAI). The grid spacing was 0.125 angstroms, which led to a grid of 15 × 15 × 15 angstroms. For each molecule, 100 docking runs were performed. The 100 docking poses generated were clustered by root mean square (RMS) difference with a cutoff value of 0.5 angstroms for each case. The top-ranked pose of the most populated clusters was retained and further analyzed with the Discovery Studio Visualizer suite (v21.1.0). β-lactose docking was performed as indicated for D-galactose, but using a grid of 124 × 124 × 124 points and a grid spacing of 0.180 angstroms, resulting in a grid of 22.32 × 22.32 × 22.32 angstroms. The grid was centered on the centroid of the pyranosic rings of lactose at the 1α or 2γ sites of the ricin structure. The 100 docking poses generated were compared with those obtained for D-galactose, and a pose was chosen that (coinciding with the best solution of docking with D-galactose) had a lower binding energy.

#### 5.3.15. Cell Viability and DNA Fragmentation Analyses

Cell viability was determined using a colorimetric assay based on the cleavage of the tetrazolium salt WST-1 to formazan by mitochondrial dehydrogenases in viable cells. First, 3 × 10^3^ COLO 320 or HeLa cells in 0.1 mL of medium were seeded in 96-well plates and incubated at 37 °C under 5% CO_2_ in the absence or the presence of RIP for 48 h. Next, the cells were incubated for another 2 h with 10 μL/well of the cell proliferation reagent WST-1 at 37 °C under 5% CO_2_. The absorbance of the samples was measured using a microtiter plate reader set at 450 nm with 620 nm as reference (ELISA reader Multiskan). The absorbance of cultures without cells was subtracted as background. For the DNA fragmentation analysis, COLO 320 cells (1 × 10^6^/plate) were incubated for 72 h in the presence of 40 nM nigrin l. After treatment, cells were harvested by centrifugation (1000*× g* for 5 min). The pellets were lysed in 50 mM Tris–HCl, pH 8.0, containing 10 mM EDTA and 0.5% SDS, and the DNA was isolated following the instruction of the Genomic Prep Cells and Tissue DNA Isolation Kit (GE Healthcare, Barcelona, Spain). DNA electrophoresis was carried out in 1.8% agarose gels using TBE buffer (0.089 M Tris, 0.089 M boric acid, 2 mM EDTA, pH 8.0), and 4.0 μg of DNA was electrophoresed and stained with GelRed (Biotium Inc., Hayward, CA, USA) and visualized with an ultraviolet lamp.

## Figures and Tables

**Table 1:** Inhibition of the hemagglutination activity of nigrin l, SNAlm, and SNAld by sugars.

	Minimum Concentration Inhibiting Hemagglutination (mM)		
Carbohydrates ^l^	Nigrin l	SNAlm	SNAld
D-galactose	50	6.25	100
Lactose	12.5	1.56	25

^1^ No inhibition of hemagglutination at the maximum sugar concentration tested (200 mM) was observed with the following sugars: D-glucose, D-fructose, D-mannose, and L-fucose.

## Data Availability

Data are available upon request; please contact the contributing authors.

## Supplementary Materials

The following supporting information can be downloaded at: https://www.mdpi.com/article/10.3390/toxins14090611/s1. Table S1: Amino acid sequences of tryptic peptides from SNAlm obtained by high-resolution nanoLC–tandem mass spectrometry and mapped on SNAlm (AC: AAN86132). Table S2: Amino acid sequences of tryptic peptides from SNAld obtained by high-resolution nanoLC–tandem mass spectrometry and mapped on SNAld (AC: AAN86131). Table S3: Amino acid sequences of tryptic peptides from nigrin l obtained by high-resolution nanoLC–tandem mass spectrometry and mapped on nigrin l (AC: AAN86130). Table S4: Amino acid sequences of tryptic peptides from nigrin-RP1 obtained by high-resolution nanoLC–tandem mass spectrometry and mapped on SNLRP2 (AC: AAC49672). Table S5: Amino acid sequences of tryptic peptides from nigrin-RP2 obtained by high-resolution nanoLC–tandem mass spectrometry and mapped on SNLRP2 (AC: AAC49672). Table S6: Amino acid sequences of tryptic peptides from nigrin-RP3 obtained by high-resolution nanoLC–tandem mass spectrometry and mapped on SNLRP1 (AC: AAC49673). Table S7: Inhibition of the hemagglutination activity of nigrin l, SNAlm, and SNAld by sugars compared with the estimated free energy of binding to the sugar binding sites. Figure S1: Effect of nigrin l, nigrin-RPs 1–4, and nigritin l on protein synthesis. Figure S2: Representative MS/MS fragmentation spectra of SNAlm peptides mapped on the protein SNAlm (AC: AAN86132). Figure S3: Representative MS/MS fragmentation spectra of SNAld peptides mapped on the protein SNAld (AC: AAN86131). Figure S4: Representative MS/MS fragmentation spectra of nigrin l peptides. Figure S5: Representative MS/MS fragmentation spectra of nigrin-RP1 peptides. Figure S6: Representative MS/MS fragmentation spectra of nigrin-RP2 peptides. Figure S7: Representative MS/MS fragmentation spectra of nigrin-RP3 peptides. Figure S8: Comparison of the binding of D-galactose and lactose to the 1α site of nigrin l, SNAlm, and SNAld. Figure S9: Comparison of the binding of D-galactose and lactose to the 2γ site of nigrin l, SNAlm, and SNAld. Figure S10: Structure of the SNAld dimer with D-galactose bound to 1α sites.
