# Supplementary material for: Structure and Biological Properties of Ribosome-Inactivating Proteins and Lectins from Elder (Sambucus nigra L.) Leaves
Source: Toxins (Basel). 2022 Sep 1;14(9):611. doi: 10.3390/toxins14090611 (PMC9503024; doi:10.3390/toxins14090611)
Supplement: Supplementary file 1 [file toxins-14-00611-s001.zip › toxins-1866353-supplementary.pdf]

Supplementary Materials: Structure and biological properties of ribosome-inactivating proteins and lectins from elder (*Sambucus nigra* L.) leaves

Rosario Iglesias, Rosita Russo, Nicola Landi, Mariangela Valletta, Angela Chambery, Antimo Di Maro, Andrea Bolognesi, José M. Ferreras and Lucía Citores

Table S1. Amino acid sequences of tryptic peptides from SNAlm obtained by high-resolution nanoLC-Tandem Mass Spectrometry and mapped on SNAlm (AC: AAN86132). Sequence, number of missed cleavages sites (MC), experimental masses of precursor ions, charge state, experimental and theoretical molecular weights of peptides (MH^{+}) together with mass accuracies and retention times are reported. m = oxidized methionine; c = carbamidomethyl cysteine.

Sequence	# Missed Cleavages	Charge	*m/z* [Da]	MH+ [Da]	Theo. MH+ [Da]	DeltaM [ppm]	RT [min]
[-].DGEPITGNIVGR.[D]	0	2	614.320	1227.632	1227.633	-0.640	31.364
[-].DGEPITGNIVGRDGLcVDVR.[N]	1	3	714.691	2142.059	2142.061	-0.950	36.009
[K].cQGLPSQR.[W]	0	2	473.232	945.456	945.457	-0.900	20.582
[K].SKLVMDVK.[S]	1	2	460.267	919.527	919.528	-1.250	22.522
[K].SKLVmDVK.[S]	1	2	468.265	935.522	935.523	-0.950	22.486
[K].SSNVSLR.[E]	0	2	381.708	762.410	762.410	-1.110	19.610
[KR].DLIIILK.[C]	0	2	414.283	827.560	827.560	-0.470	40.489
[R].DGLcVDVR.[ND]	0	2	467.227	933.446	933.446	-0.120	30.447
[R].EIIIYPATGRPNQQWVTQVLPS.[-]	0	2	1255.672	2510.336	2510.340	-1.480	42.248
[R].EIIIYPATGRPNQQWVTQVLPS.[-]	0	3	837.451	2510.337	2510.340	-1.140	42.462
[R].GLcVTTNGYNSR.[D]	0	2	671.314	1341.620	1341.622	-1.230	26.564
[R].GLcVTTNGYNSRDLIIILK.[C]	1	3	717.392	2150.161	2150.164	-1.220	38.380
[R].NGYDTDGTPIQLWPcGTQR.[N]	0	2	1089.996	2178.984	2178.987	-1.540	39.070
[R].NGYDTDGTPIQLWPcGTQR.[N]	0	3	727.000	2178.985	2178.987	-1.180	39.068
[R].NQQWTFHTDDTIR.[S]	0	3	554.594	1661.766	1661.767	-0.200	30.596
[R].NQQWTFHTDDTIR.[S]	0	2	831.386	1661.764	1661.767	-1.550	30.377
[R].TIRVNSNR.[GD]	1	2	480.273	959.538	959.538	-0.290	18.069
[R].WFFNSDGAIVNPK.[S]	0	2	747.872	1494.736	1494.738	-1.140	39.435

Table S2. Amino acid sequences of tryptic peptides from SNAld obtained by high-resolution nanoLC-Tandem Mass Spectrometry and mapped on SNAld (AC: AAN86131). Sequence, number of missed cleavages sites (MC), experimental masses of precursor ions, charge state, experimental and theoretical molecular weights of peptides (MH+) together with mass accuracies and retention times are reported. m = oxidized methionine; c = carbamidomethyl cysteine.

Sequence	# Missed Cleavages	Charge	*m/z* [Da]	MH+ [Da]	Theo. MH+ [Da]	DeltaM [ppm]	RT [min]
[-].DGEPFTR.[H]	0	2	411.193	821.379	821.379	0.180	25.837
[K].VWMENcVSTSVQQQWVLFGDR.[T]	0	3	857.069	2569.193	2569.196	-1.370	44.472
[K].WRTEVLPS.[-]	1	2	494.266	987.525	987.526	-0.810	31.638
[R].DGLcVDAR.[T]	0	2	453.210	905.414	905.415	-1.080	28.326
[R].DLcLTSSGHVSK.[D]	0	3	435.215	1303.631	1303.631	0.000	25.055
[R].DLcLTSSGHVSK.[D]	0	2	652.318	1303.630	1303.631	-1.160	25.067
[R].EIIIFPATGNPNQK.[W]	0	2	771.419	1541.830	1541.832	-1.280	36.609
[R].EIIIFPATGNPNQK.[W]	0	3	514.616	1541.832	1541.832	0.140	36.636
[R].TGcDIQLWPcGSQTSQQWTFYEDGTIR.[S]	0	3	1078.812	3234.420	3234.426	-1.590	43.809

Table S3. Amino acid sequences of tryptic peptides from nigrin l obtained by high-resolution nanoLC-Tandem Mass Spectrometry and mapped on nigrin l (AC: AAN86130). Sequence, number of missed cleavages sites (MC), experimental masses of precursor ions, charge state, experimental and theoretical molecular weights of peptides (MH+) together with mass accuracies and retention times are reported. m = oxidized methionine; c = carbamidomethyl cysteine.

Sequence	# Missed Cleavages	Charge	*m/z* [Da]	MH+ [Da]	Theo. MH+ [Da]	Delta M [ppm]	RT [min]
[-].IDYPSVSFNLDGAK.[S]	0	2	763.381	1525.755	1525.753	0.830	38.891
[-].IDYPSVSFNLDGAK.[S]	0	3	509.256	1525.753	1525.753	-0.120	38.738
[K].DATEVQK.[S]	0	2	395.701	790.395	790.394	0.500	17.962
[K].ITGIAILLFR.[C]	0	2	558.860	1116.713	1116.714	-0.570	45.253
[K].QNTLSFTGNYDNLETAANTR.[R]	0	2	1115.521	2230.034	2230.037	-1.280	36.155
[K].QNTLSFTGNYDNLETAANTR.[R]	0	3	744.018	2230.040	2230.037	1.400	36.210
[K].QNTLSFTGNYDNLETAANTRR.[E]	1	3	796.050	2386.135	2386.138	-1.400	33.312
[K].SATYRDFLSNLR.[K]	1	3	481.584	1442.738	1442.739	-0.680	35.243
[K].SATYRDFLSNLRK.[T]	2	3	524.282	1570.833	1570.834	-0.600	31.647
[K].SNLFVGTK.[Q]	0	2	433.243	865.478	865.478	0.110	27.928
[K].TVATGTYEVNGLPVLR.[R]	0	2	845.461	1689.915	1689.917	-1.140	37.167
[K].TVATGTYEVNGLPVLR.[R]	0	3	563.977	1689.916	1689.917	-0.350	37.182
[K].TVATGTYEVNGLPVLRR.[E]	1	3	616.011	1846.017	1846.018	-0.340	33.132
[K].TVATGTYEVNGLPVLRR.[E]	1	2	923.511	1846.015	1846.018	-1.460	33.160
[R].DFLSNLR.[K]	0	2	432.733	864.458	864.457	0.410	37.969
[R].DFLSNLRK.[T]	1	2	496.780	992.552	992.552	-0.060	32.419
[R].ESEVQVK.[SN]	0	2	409.716	818.426	818.425	0.270	18.351
[R].ESIELGPSPLDGAITSLYHGDSVAR.[S]	0	3	862.101	2584.289	2584.289	-0.030	41.678
[R].ESIELGPSPLDGAITSLYHGDSVAR.[S]	0	2	1292.646	2584.286	2584.289	-1.260	41.467
[R].ESIELGPSPLDGAITSLYHGDSVAR.[S]	0	4	646.827	2584.287	2584.289	-0.840	41.515
[R].KTVATGTYEVNGLPVLR.[R]	1	2	909.508	1818.009	1818.012	-1.730	32.986

Table S3. Continuation

Sequence	# Missed Cleavages	Charge	*m*/*z* [Da]	MH+ [Da]	Theo. MH+ [Da]	Delta M [ppm]	RT [min]
[R].KTVATGTYEVNGLPVLR.[R]	1	3	606.675	1818.011	1818.012	-0.560	32.924
[R].KTVATGTYEVNGLPVLRR.[E]	2	3	658.709	1974.111	1974.113	-0.900	29.848
[R].KTVATGTYEVNGLPVLRR.[E]	2	4	494.284	1974.113	1974.113	-0.260	29.791
[R].LVDNFEELYK.[I]	0	2	635.322	1269.636	1269.636	-0.280	37.020
[R].LVDNFEELYKITGIAILLFR.[C]	1	3	789.781	2367.329	2367.332	-1.290	53.412
[R].RESEVQVK.[SN]	1	2	487.767	974.527	974.527	0.940	17.958
[R].RESIELGPSPLDGAITSLYHGDSVAR.[S]	1	4	685.852	2740.388	2740.390	-0.810	38.342
[R].RESIELGPSPLDGAITSLYHGDSVAR.[S]	1	3	914.134	2740.387	2740.390	-1.180	38.375
[R].SLLVVIQMVSEAAR.[F]	0	2	758.432	1515.856	1515.856	-0.290	48.132
[R].SLLVVIQMVSEAAR.[F]	0	3	505.957	1515.856	1515.856	-0.100	47.891
[R].SLLVVIQmVSEAAR.[F]	0	2	766.428	1531.848	1531.851	-2.070	47.933
[R].YIEQEVR.[R]	0	2	468.742	936.478	936.479	-1.060	22.831
[-].DGETcTLR.[T]	0	2	476.214	951.420	951.420	-0.310	23.356
[-].DGETcTLRTSFTR.[N]	1	3	515.244	1543.717	1543.717	0.010	26.308
[K].cQGLPSQR.[W]	0	2	473.232	945.457	945.457	-0.190	21.489
[K].EMcLQSNGENNGVWMEDcEATSLQQ QWALYGDR.[T]	0	4	980.664	3919.635	3919.641	-1.550	43.644
[K].EMcLQSNGENNGVWMEDcEATSLQQ QWALYGDR.[T]	0	3	1307.218	3919.638	3919.641	-0.880	43.631
[KR].DLIIILK.[C]	0	2	414.284	827.560	827.560	-0.100	41.521
[R].DGLcVDVR.[ND]	0	2	467.226	933.446	933.446	-0.250	32.096
[R].EIIIFPATGNPNQQWVTQVLPS.[-]	0	3	818.100	2452.285	2452.287	-0.770	50.168
[R].EIIIFPATGNPNQQWVTQVLPS.[-]	0	2	1226.646	2452.284	2452.287	-1.170	50.180
[R].GLcVTTNGYNSK.[D]	0	2	657.311	1313.614	1313.615	-1.040	26.147
[R].GLcVTTNGYNSKDLIIILK.[C]	1	3	708.057	2122.157	2122.158	-0.300	38.226
[R].NIVGRDGLcVDVR.[N]	1	3	491.593	1472.763	1472.764	-0.350	30.559
[R].NIVGRDGLcVDVR.[N]	1	2	736.885	1472.762	1472.764	-1.150	30.621
[R].TILLLEDNIYAASQGWTVTNNVKPIV ASIVGYK.[E]	0	4	898.492	3590.945	3590.947	-0.530	48.083
[R].TILLLEDNIYAASQGWTVTNNVKPIV ASIVGYK.[E]	0	3	1197.653	3590.945	3590.947	-0.400	48.090
[R-].TSFTRNIVGR.[D]	1	3	384.215	1150.632	1150.633	-0.720	25.162
[R].WFFNSDGAIVNPK.[S]	0	2	747.872	1494.737	1494.738	-0.730	39.687
[R].WFFNSDGAIVNPK.[S]	0	3	498.917	1494.737	1494.738	-0.540	39.516
[R].WFFNSDGAIVNPK.[S]	0	2	747.872	1494.737	1494.738	-0.730	39.687
[R].WFFNSDGAIVNPK.[S]	0	3	498.917	1494.737	1494.738	-0.540	39.516

Table S4. Amino acid sequences of tryptic peptides from nigrin-RP1 obtained by high-resolution nanoLC-Tandem Mass Spectrometry and mapped on SNLRP2 (AC: AAC49672). Sequence, number of missed cleavages sites (MC), experimental masses of precursor ions, charge state, experimental and theoretical molecular weights of peptides (MH+) together with mass accuracies and retention times are reported. m = oxidized methionine; c = carbamidomethyl cysteine.

Sequence	# Missed Cleavages	Charge	*m/z* [Da]	MH+ [Da]	Theo. MH+ [Da]	DeltaM [ppm]	RT [min]
[K].ALLVVIQMVSEAAR.[F]	0	2	750.433	1499.860	1499.861	-1.240	46.629
[K].ALLVVIQMVSEAAR.[F]	0	3	500.625	1499.860	1499.861	-0.640	46.647
[K].ESTDIEK.[K]	0	2	411.198	821.388	821.389	-0.580	18.236
[K].FTGNYNSLEQQAGDRR.[E]	1	3	619.294	1855.867	1855.868	-0.770	27.300
[K].KNLFVDTK.[Q]	1	2	482.776	964.546	964.546	-0.710	25.708
[K].NLFVDTK.[Q]	0	2	418.729	836.451	836.451	-0.320	30.230
[K].NLFVDTKQENLK.[F]	1	3	483.596	1448.773	1448.774	-0.680	29.492
[K].SYTSLDLPVLKPQVPVENR.[F]	0	3	719.063	2155.174	2155.176	-0.870	37.751
[R].ESIPLGPNSLAQAISSLSR.[Y]	0	2	970.524	1940.041	1940.045	-2.080	46.496
[R].NFLGELQDLVTR.[K]	0	2	702.877	1404.747	1404.748	-0.910	46.478
[K].cLTNSGDSSGNYAMIYNcDTAIR.[D]	0	3	861.701	2583.090	2583.091	-0.450	36.202
[K].cTVVDVTR.[R]	0	2	475.242	949.476	949.477	-0.820	25.229
[K].DGNPVQLLScGQQSSQQWTFR.[T]	0	3	812.716	2436.133	2436.136	-1.300	41.886
[K].LVmEVR.[Q]	0	2	381.712	762.417	762.418	-0.880	27.438
[K].WVLSIDGTITHR.[L]	0	3	466.589	1397.753	1397.754	-0.760	36.821
[K].WVLSIDGTITHR.[L]	0	2	699.380	1397.752	1397.754	-0.780	36.876
[R].DGLcVDVR.[ND]	0	2	467.226	933.445	933.446	-0.780	30.643
[R].DGLcVDVRDGLAK.[D]	1	3	473.241	1417.709	1417.710	-0.860	31.058
[R].LSGLVLTAPQAAQGTTLLLQK.[N]	0	2	1062.124	2123.240	2123.243	-1.480	41.474
[R].LSGLVLTAPQAAQGTTLLLQK.[N]	0	4	531.566	2123.244	2123.243	0.060	41.525
[R].LSGLVLTAPQAAQGTTLLLQK.[N]	0	3	708.419	2123.241	2123.243	-1.140	42.003
[R].QIIIYHPTGNLNQQWITDTDPV.[-]	0	3	856.102	2566.291	2566.294	-1.220	41.724
[R].QSDVSLR.[Q]	0	2	402.714	804.421	804.421	-0.610	21.437

Table S5. Amino acid sequences of tryptic peptides from nigrin-RP2 obtained by high-resolution nanoLC-Tandem Mass Spectrometry and mapped on SNLRP2 (AC: AAC49672). Sequence, number of missed cleavages sites (MC), experimental masses of precursor ions, charge state, experimental and theoretical molecular weights of peptides (MH+) together with mass accuracies and retention times are reported. m = oxidized methionine; c = carbamidomethyl cysteine.

Sequence	# Missed Cleavages	Charge	*m/z* [Da]	MH+ [Da]	Theo. MH+ [Da]	DeltaM [ppm]	RT [min]
[-].APPNYPSVSLK.[M]	0	2	586.82	1172.63	1172.63	-1.22	30.99
[K].ALLVVIQMVSEAAR.[F]	0	2	750.43	1499.86	1499.86	-1.16	46.79
[K].ALLVVIQMVSEAAR.[F]	0	3	500.63	1499.86	1499.86	-0.64	46.83
[K].FTGNYNSLEQQAGDRR.[E]	1	3	619.29	1855.87	1855.87	-0.77	27.34
[K].NLFVDTK.[Q]	0	2	418.73	836.45	836.45	-1.42	31.00
[K].SYTSLDLPVLKPQVPVENR.[F]	0	3	719.06	2155.17	2155.18	-0.70	37.85
[R].ESIPLGPNSLAQAISSLSR.[Y]	0	2	970.52	1940.04	1940.04	-1.45	46.54
[R].ESIPLGPNSLAQAISSLSR.[Y]	0	3	647.35	1940.04	1940.04	-0.95	46.60
[R].NFLGELQDLVTR.[K]	0	2	702.88	1404.75	1404.75	-1.00	46.60
[K].cTVVDVTR.[R]	0	2	475.24	949.48	949.48	-0.11	25.43
[K].DGNPVQLLScGQQSSQQWTFR.[T]	0	3	812.72	2436.13	2436.14	-0.55	41.87
[K].LVmEVR.[Q]	0	2	381.71	762.42	762.42	-0.96	27.90
[K].WVLSIDGTITHR.[L]	0	3	466.59	1397.75	1397.75	-0.63	37.42
[K].WVLSIDGTITHR.[L]	0	2	699.38	1397.75	1397.75	-0.69	36.94
[R].ANTQNNPLWLEDcVLNR.[TR]	0	3	686.33	2056.99	2056.99	-0.56	41.17
[R].DGLcVDVR.[ND]	0	2	467.23	933.45	933.45	-0.32	30.85
[R].DGLcVDVRDGLAK.[D]	1	3	473.24	1417.71	1417.71	-0.99	31.15
[R].DGLcVDVRDGLAK.[D]	1	2	709.36	1417.71	1417.71	-0.18	31.88
[R].LSGLVLTAPQAAQGTTLLLQK.[N]	0	3	708.42	2123.24	2123.24	-0.28	41.72
[R].LSGLVLTAPQAAQGTTLLLQK.[N]	0	2	1062.12	2123.24	2123.24	-1.71	41.49
[R].LSGLVLTAPQAAQGTTLLLQK.[N]	0	4	531.57	2123.24	2123.24	0.52	41.52
[R].QIIIYHPTGNLNQQWITDTDPV.[-]	0	3	856.10	2566.29	2566.29	0.21	41.72
[R].QIIIYHPTGNLNQQWITDTDPV.[-]	0	2	1283.65	2566.29	2566.29	-0.81	41.72
[R].QSDVSLR.[Q]	0	2	402.71	804.42	804.42	-0.53	22.00

Table S6. Amino acid sequences of tryptic peptides from nigrin-RP3 obtained by high-resolution nanoLC-Tandem Mass Spectrometry and mapped on SNLRP1 (AC: AAC49673). Sequence, number of missed cleavages sites (MC), experimental masses of precursor ions, charge state, experimental and theoretical molecular weights of peptides (MH+) together with mass accuracies and retention times are reported. c = carbamidomethyl cysteine; m = oxidized methionine; c = carbamidomethyl cysteine.

Sequence	# Missed Cleavages	Charge	*m/z* [Da]	MH+ [Da]	Theo. MH+ [Da]	DeltaM [ppm]	RT [min]
[K].ALLVVIQMVSEAAR.[F]	0	3	500.625	1499.86	1499.861	-0.76	46.7279
[R].NFLGELQDLVTR.[K]	0	2	702.878	1404.747	1404.748	-0.82	46.7094
[K].ALLVVIQMVSEAAR.[F]	0	2	750.434	1499.86	1499.861	-0.92	46.673
[-].ATPPNYPSVSLK.[M]	0	2	637.343	1273.678	1273.679	-0.32	30.9333
[K].cTVVDVTR.[R]	0	2	475.242	949.477	949.477	0.340	25.303
[K].cTVVDVTRR.[I]	1	2	553.292	1105.577	1105.578	-0.850	22.847
[K].DGNPVQLLScGQQSSQQWTFR.[T]	0	2	1218.570	2436.133	2436.136	-1.220	41.845
[K].DGNPVQLLScGQQSSQQWTFR.[T]	0	3	812.717	2436.135	2436.136	-0.400	42.024
[K].LVmEVR.[Q]	0	2	381.712	762.417	762.418	-0.960	27.663
[K].WVLSIDGTITHR.[L]	0	3	466.589	1397.753	1397.754	-0.170	36.921
[K].WVLSIDGTITHR.[L]	0	2	699.380	1397.753	1397.754	-0.690	36.713
[R].DGLcVDVR.[ND]	0	2	467.226	933.446	933.446	-0.250	30.603
[R].DGLcVDVRDGLAK.[D]	1	3	473.241	1417.709	1417.710	-0.790	31.039
[R].ISGRDGLcVDVR.[D]	1	3	449.565	1346.682	1346.685	-1.990	27.826
[R].LSGLVLTAPQAAQGTTLLLQK.[N]	0	3	708.419	2123.241	2123.243	-0.970	41.401
[R].LSGLVLTAPQAAQGTTLLLQK.[N]	0	2	1062.124	2123.240	2123.243	-1.600	41.432
[R].LSGLVLTAPQAAQGTTLLLQK.[N]	0	4	531.566	2123.244	2123.243	0.170	41.476
[R].QIIIYHPTGNLNQQWITDTDPV.[-]	0	3	856.102	2566.291	2566.294	-1.000	41.710
[R].QIIIYHPTGNLNQQWITDTDPV.[-]	0	2	1283.649	2566.291	2566.294	-1.190	41.752
[R].QSDVSLR.[Q]	0	2	402.714	804.421	804.421	-0.530	21.745
[R].SLcVSHSSSDLIIILK.[C]	0	3	591.326	1771.962	1771.962	0.040	37.352
[R].SLcVSHSSSDLIIILK.[C]	0	2	886.484	1771.960	1771.962	-1.060	37.371
[R].TEQMWALYGDGTIR.[V]	0	2	820.890	1640.772	1640.774	-0.960	39.382
[R].TEQmWALYGDGTIR.[V]	0	2	828.887	1656.766	1656.769	-1.340	36.640
[R].TEQMWALYGDGTIR.[V]	0	3	547.596	1640.773	1640.774	-0.370	39.395
[R].TEQmWALYGDGTIR.[V]	0	3	552.928	1656.769	1656.769	0.010	36.652
[R].WVFNTNGTISNPNAK.[L]	0	2	831.915	1662.822	1662.823	-0.680	35.480

Table S7. Inhibition of the hemagglutination activity of nigrin l, SNAlm and SNAld by sugars compared with the Estimated Free Energy of Binding to the sugar-binding sites.

D-Galactose

		Estimated Free Energy of Binding (kcal/mol)	
	Agglutination inhibition (mM)	1-alpha site	2-gamma site
Nigrina l	50	-4.54	-4.29
SNAlm	6.25	-4.79	-5.06
SNAld	100	-5.2	-4.55

Lactose

		Estimated Free Energy of Binding (kcal/mol)	
	Agglutination inhibition (mM)	1-alpha site	2-gamma site
Nigrina l	12.5	-4.15	-2.75
SNAlm	1.56	-3.93	-3.42
SNAld	25	-3.24	-2.54

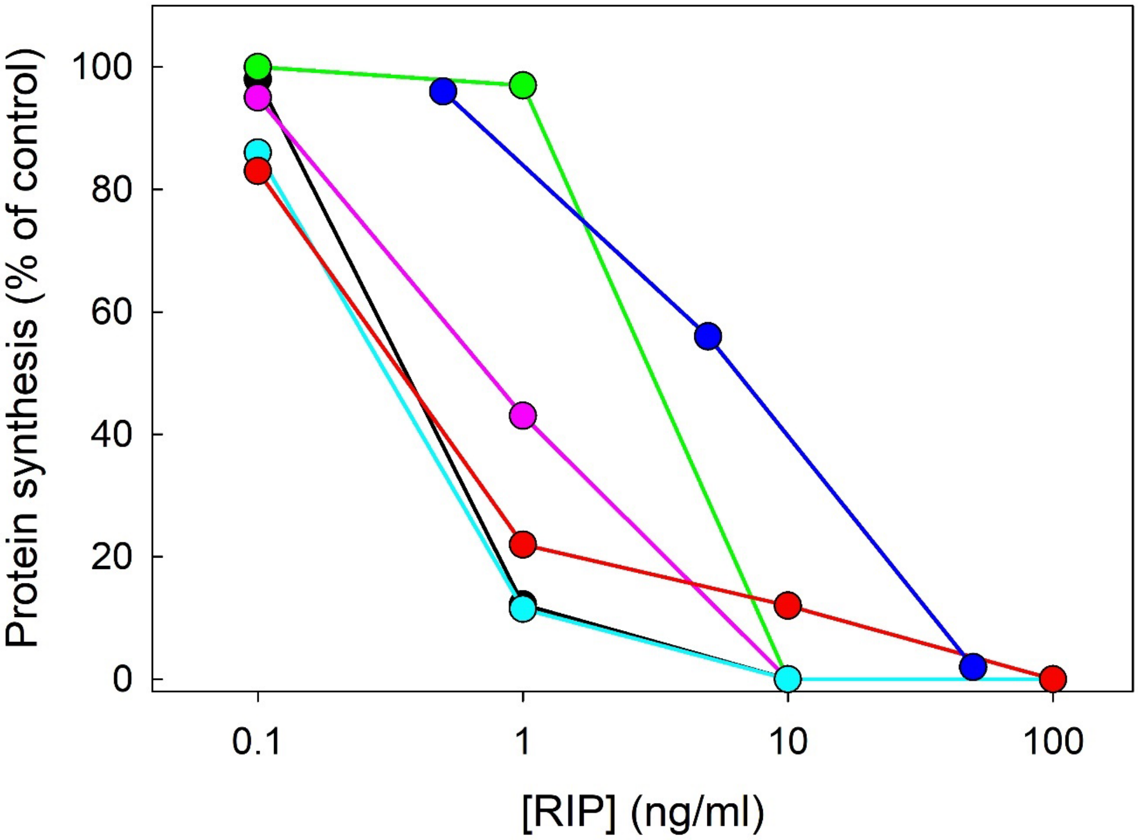

Figure S1. Effect of nigrin l, nigrin-RPs 1-4 and nigritin l on protein synthesis. Translation assays were carried out using rabbit reticulocytes lysate as a cell-free system, as indicated in Materials and methods. Symbols: black, nigrin l; pink, nigrin-RP1; green, nigrin-RP2; red, nigrin-RP3; cyan, nigrin-RP4; dark blue, nigritin l.

a

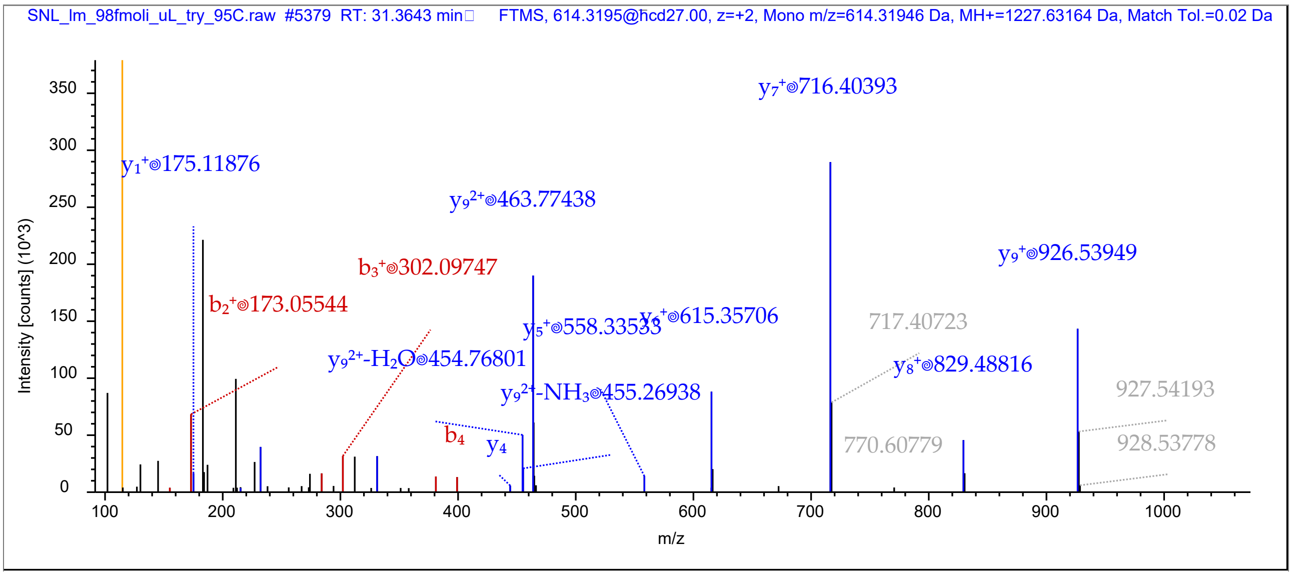

b

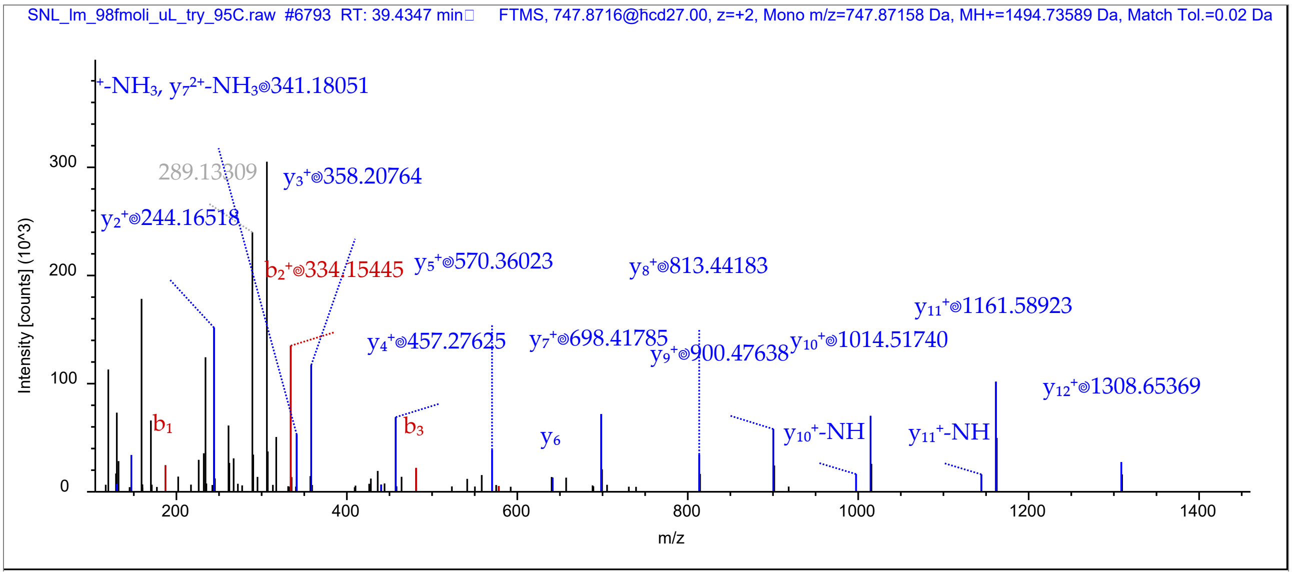

Figure S2. Representative MS/MS fragmentation spectra of SNAlm peptides mapped on the protein SNAlm (AC: AAN86132). (**a**) MS/MS fragmentation spectrum of the double charged ion at m/z 614.319, mapped on the tryptic peptide [-].DGEPITGNIVGR.[D]. (**b**) MS/MS fragmentation spectrum of the double charged ion at m/z 747.872, mapped on the tryptic peptide [R].WFFNSDGAIVNPK.[S].

a

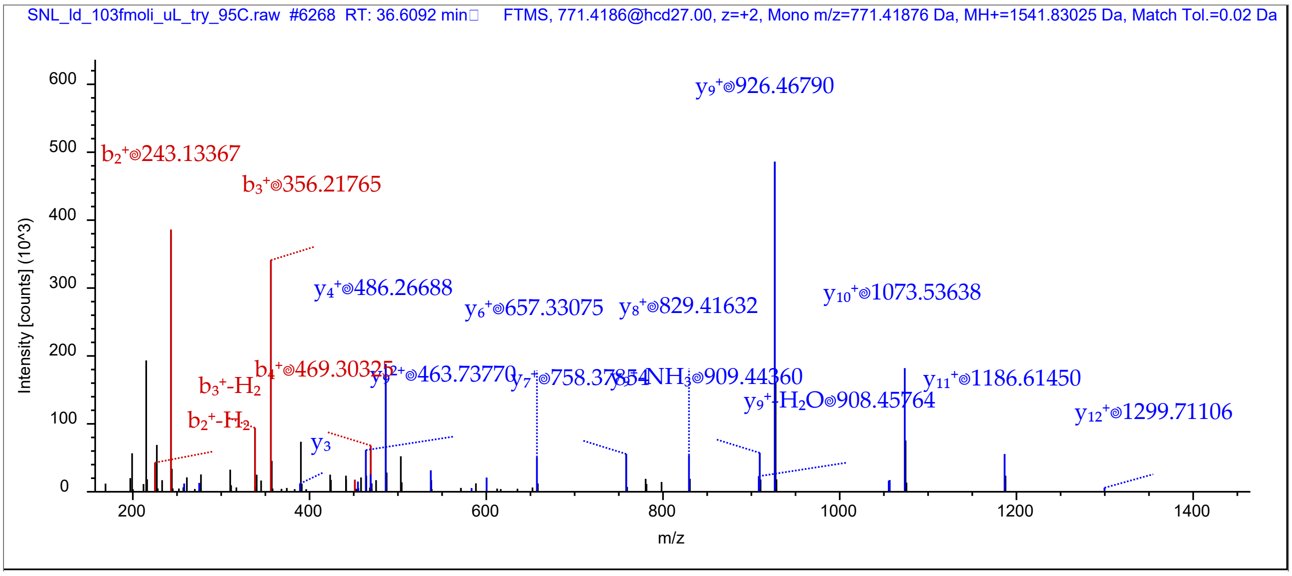

b

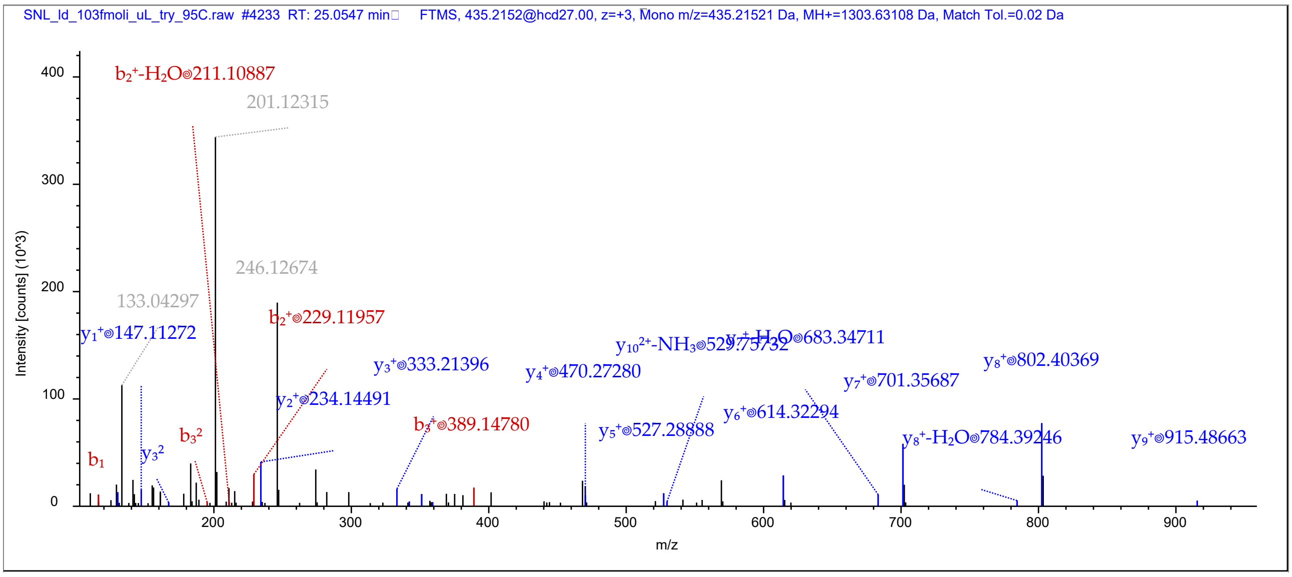

Figure S3. Representative MS/MS fragmentation spectra of SNAld peptides mapped on the protein SNAld (AC: AAN86131). (**a**) MS/MS fragmentation spectrum of the double charged ion at m/z 771.419, mapped on the tryptic peptide [R].EIIIFPATGNPNQK.[W]. (**b**) MS/MS fragmentation spectrum of the triple charged ion at m/z 435.215, mapped on the tryptic peptide [R].DLcLTSSGHVSK.[D].

a

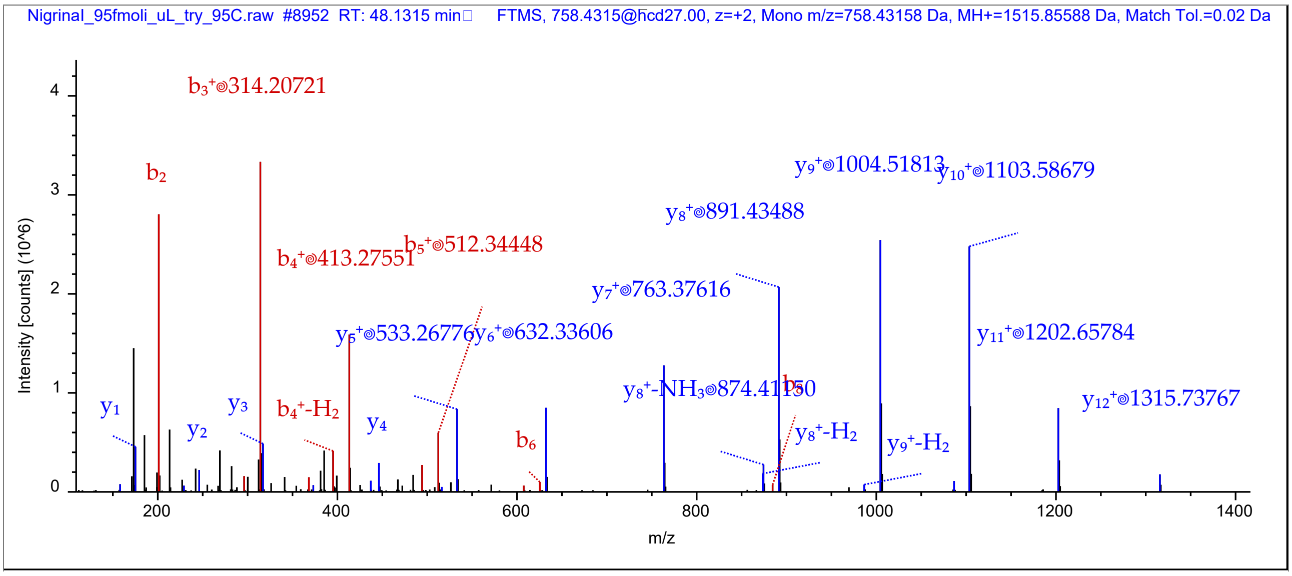

b

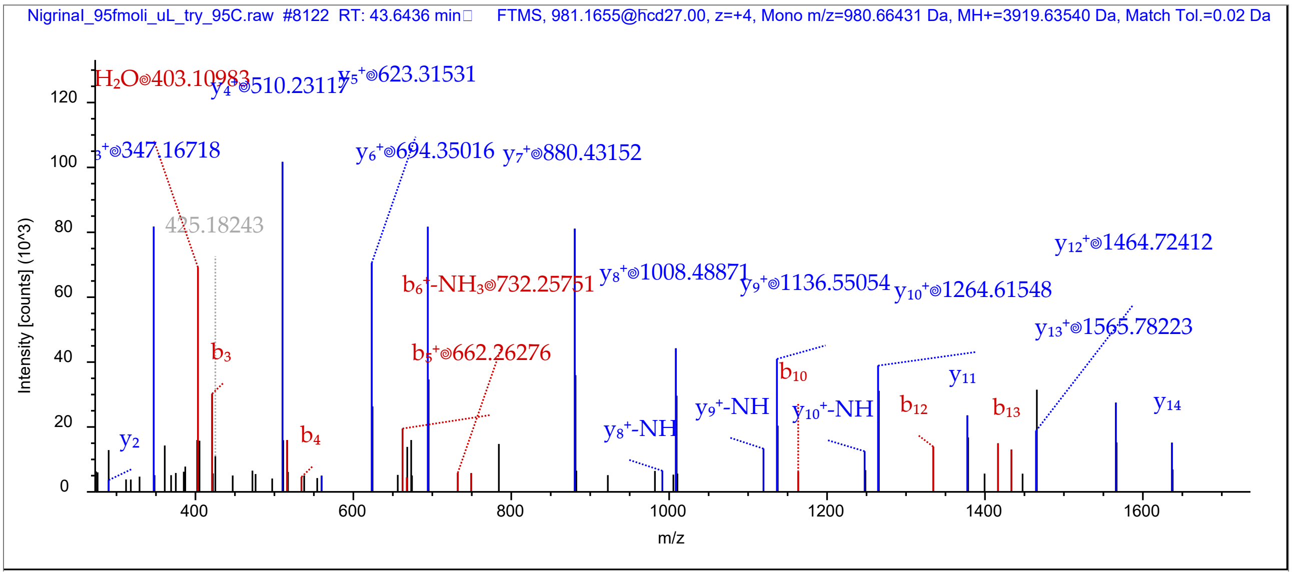

Figure S4. Representative MS/MS fragmentation spectra of nigrin 1 peptides. (**a**) MS/MS fragmentation spectrum of the double charged ion at m/z 758.432, mapped on the tryptic peptide [R].SLLVVIQMVSEAAR.[F] of protein Nigrin_1_A-chain (AC: AAN86130). (**b**) MS/MS fragmentation spectrum of the quadruple charged ion at m/z 980.664, mapped on the tryptic peptide [K].EMCLQSNGENNGVWMEDCEATSLQQQWALYGDR.[T] of the protein Nigrin 1_B-chain (AC: AAN86130).

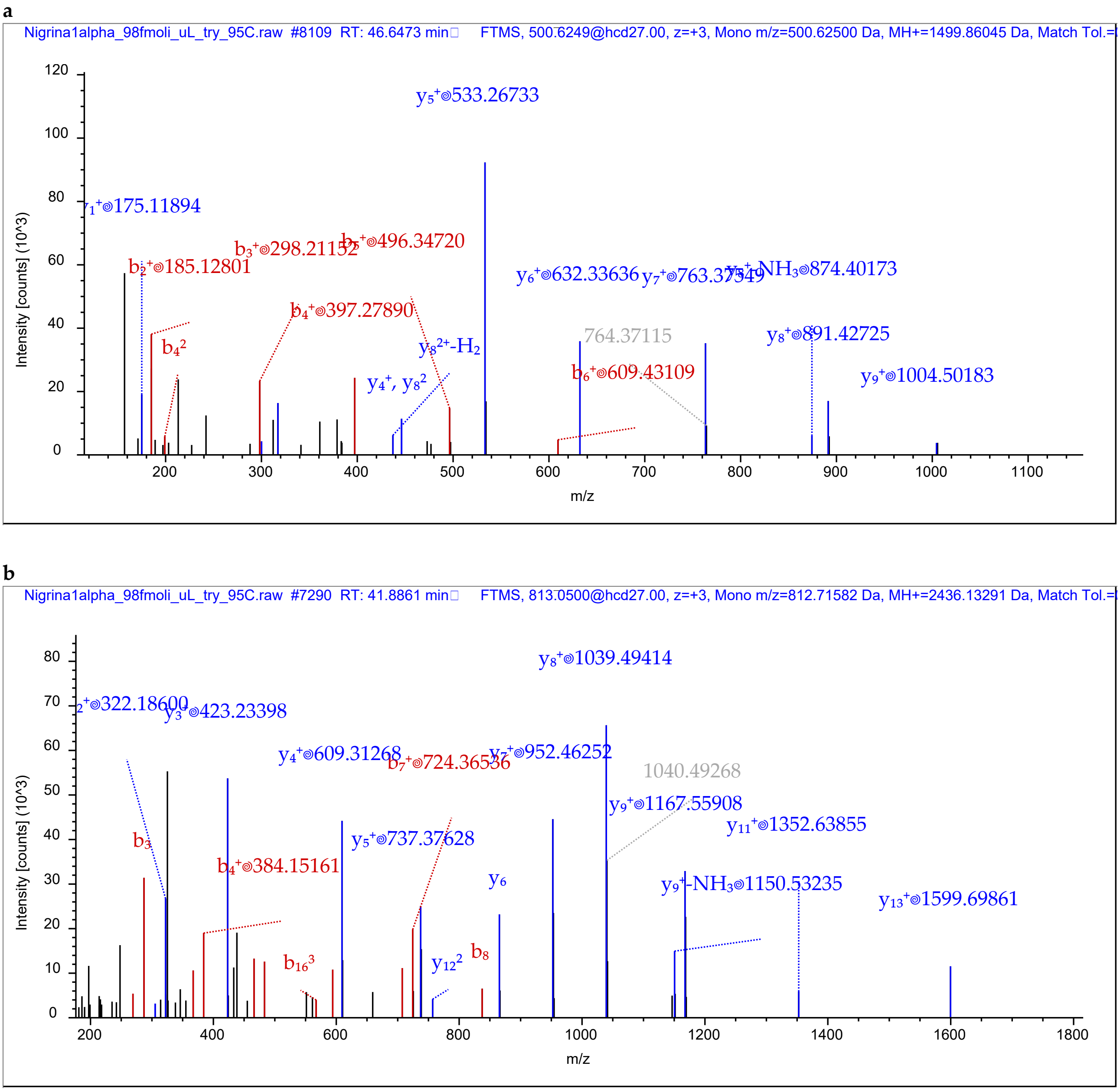

Figure S5. Representative MS/MS fragmentation spectra of nigrin-RP1 peptides. (**a**) MS/MS fragmentation spectrum of the triple charged ion at m/z 500.625, mapped on the tryptic peptide [K].ALLVVIQMVSEAAR.[F] of the protein SNLRP2_A-chain (AC: AAC49672). (**b**) MS/MS fragmentation spectrum of the triple charged ion at m/z 812.716, mapped on the tryptic peptide [K].DGNPVQLLScGQQSSQQWTFR.[T] of the protein SNLRP2_B-chain (AC: AAC49672).

a

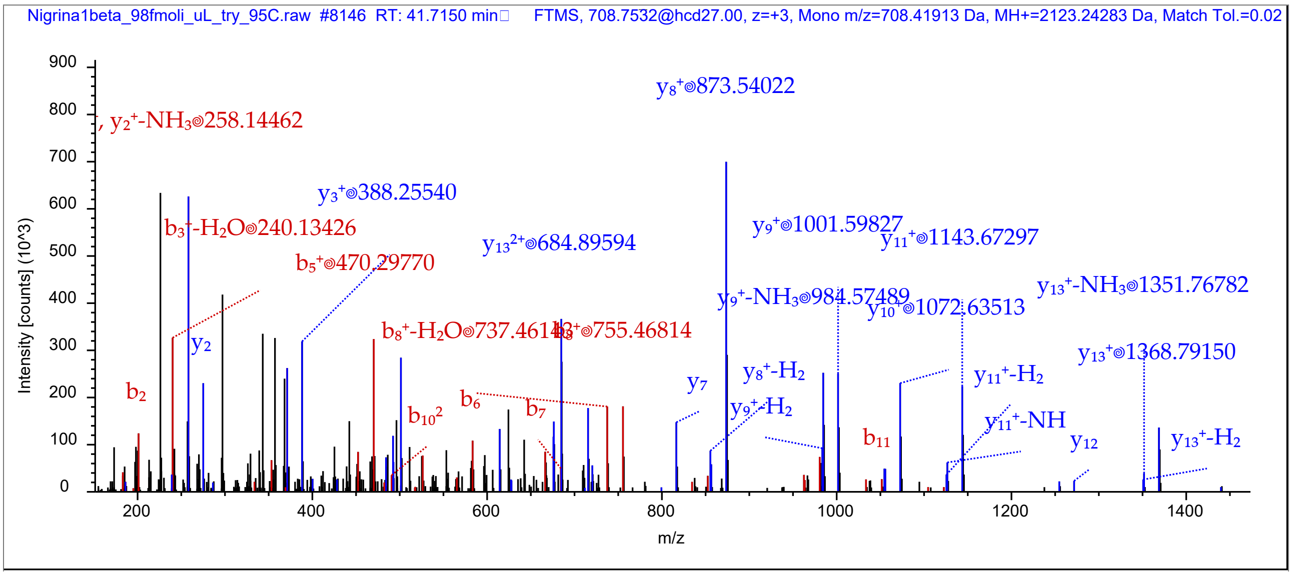

b

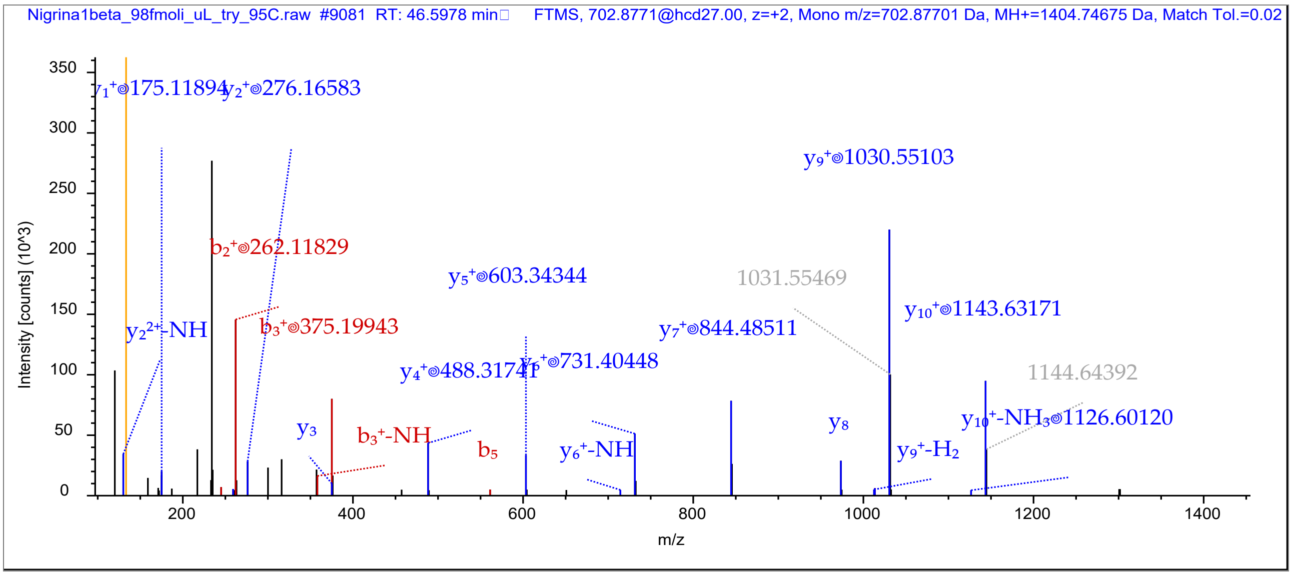

Figure S6. Representative MS/MS fragmentation spectra of nigrin-RP2 peptides. (**a**) MS/MS fragmentation spectrum of the double charged ion at m/z 702.877, mapped on the tryptic peptide [R].NFLGELQDLVTR.[K] of the protein SNLRP2_A-chain_(AC: AAC49672). (**b**) MS/MS fragmentation spectrum of the triple charged ion at m/z 708.419, mapped on the tryptic peptide [R].LSGLVLTAPQAAQGTTLLLQK.[N] of the protein SNLRP2_B-chain_ (AC: AAC49672).

a

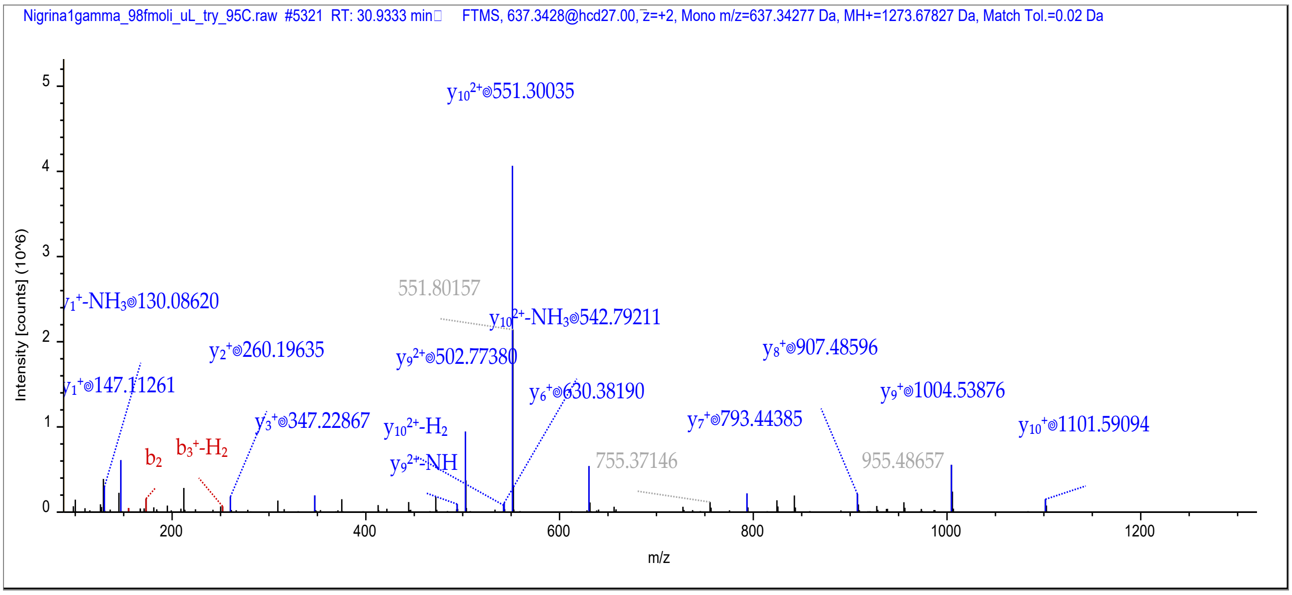

b

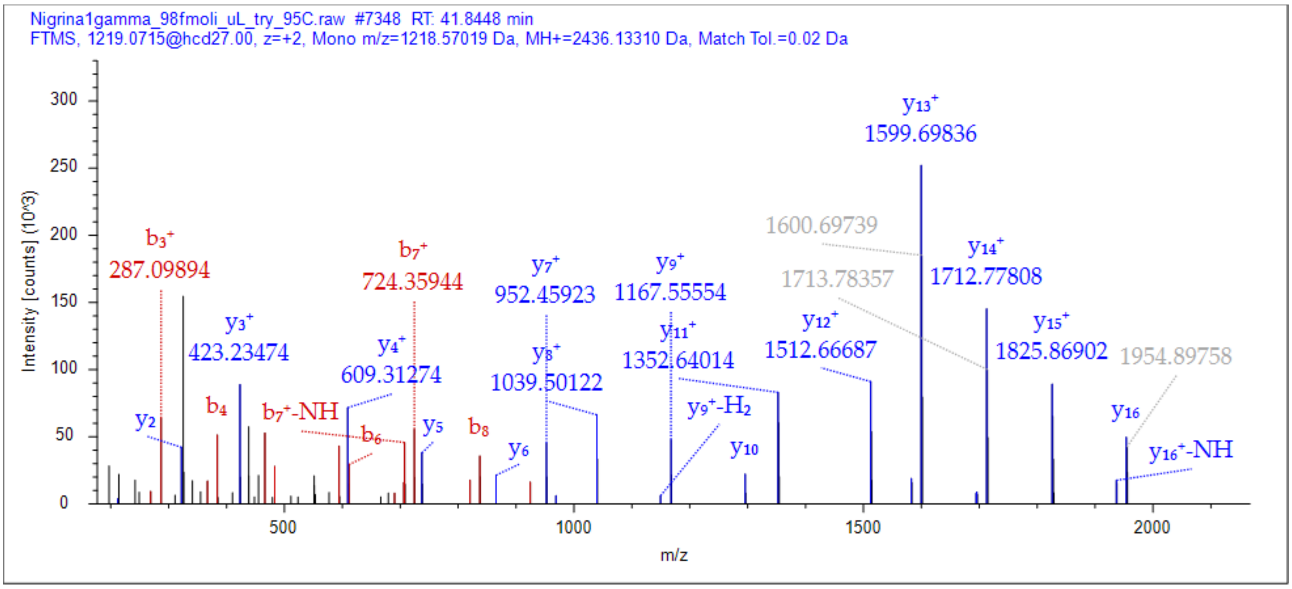

Figure S7. Representative MS/MS fragmentation spectra of nigrin-RP3 peptides. (**a**) MS/MS fragmentation spectrum of the double charged ion at m/z 637.343 from nigrin-RP3, mapped on the tryptic peptide [-].ATPPNYPSVSLK.[M] of the protein SNLRP1_A-chain (AC: AAC49673). (**b**) MS/MS fragmentation spectrum of the double charged ion at m/z 1218.570 from nigrin-RP3, mapped on the tryptic peptide [K].DGNPVQLLScGQQSSQQWTFR.[T] of the protein SNLRP1_B-chain (AC: AAC49673).

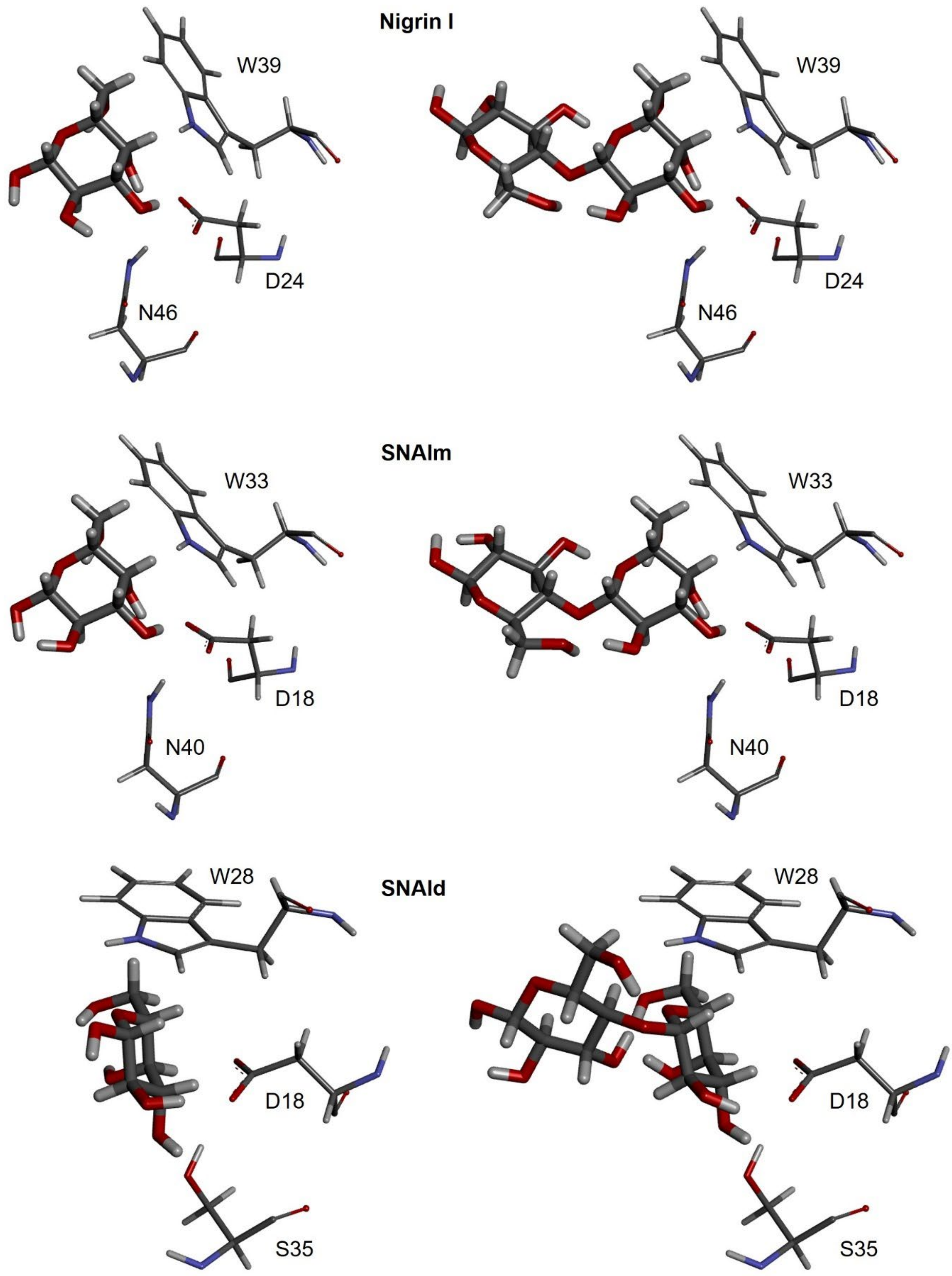

Figure S8. Comparison of the binding of D-galactose and lactose to the 1α site of nigrin l, SNAlm and SNAld. The three-dimensional models of the 1α site from either nigrin l, SNAlm and SNAld complexed with β-D-galactopyranose (left, thick sticks) or lactose (right, thick sticks) are represented. Some amino acids that bind the galactose molecule by either C-H-π interactions or hydrogen bonds are represented by thin sticks.

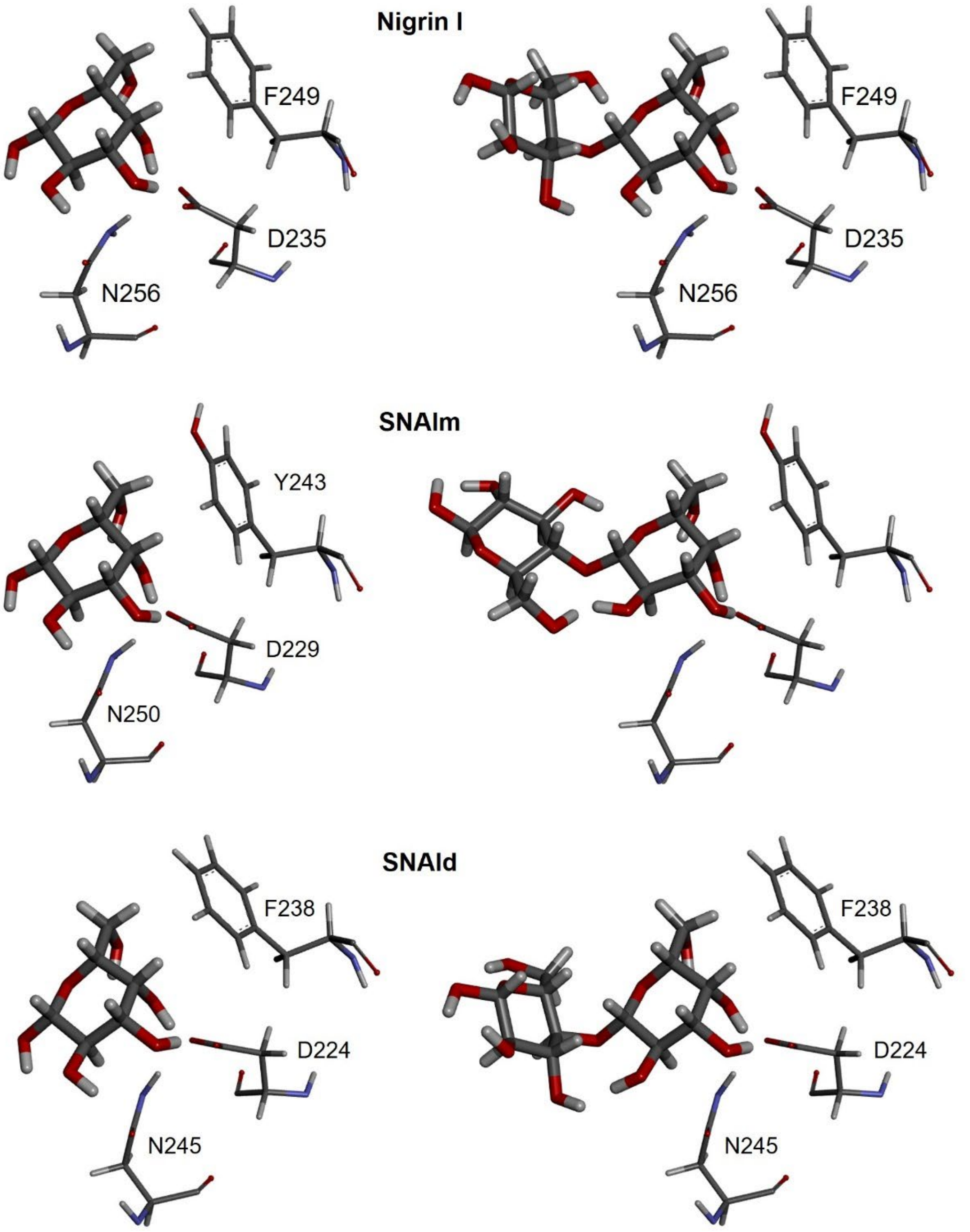

Figure S9. Comparison of the binding of D-galactose and lactose to the 2γ site of nigrin l, SNAlm and SNAld. The three-dimensional models of the 2γ site from either nigrin l, SNAlm and SNAld complexed with β-D-galactopyranose (left, thick sticks) or lactose (right, thick sticks) are represented. Some amino acids that bind the galactose molecule by either C-H-π interactions or hydrogen bonds are represented by thin sticks.

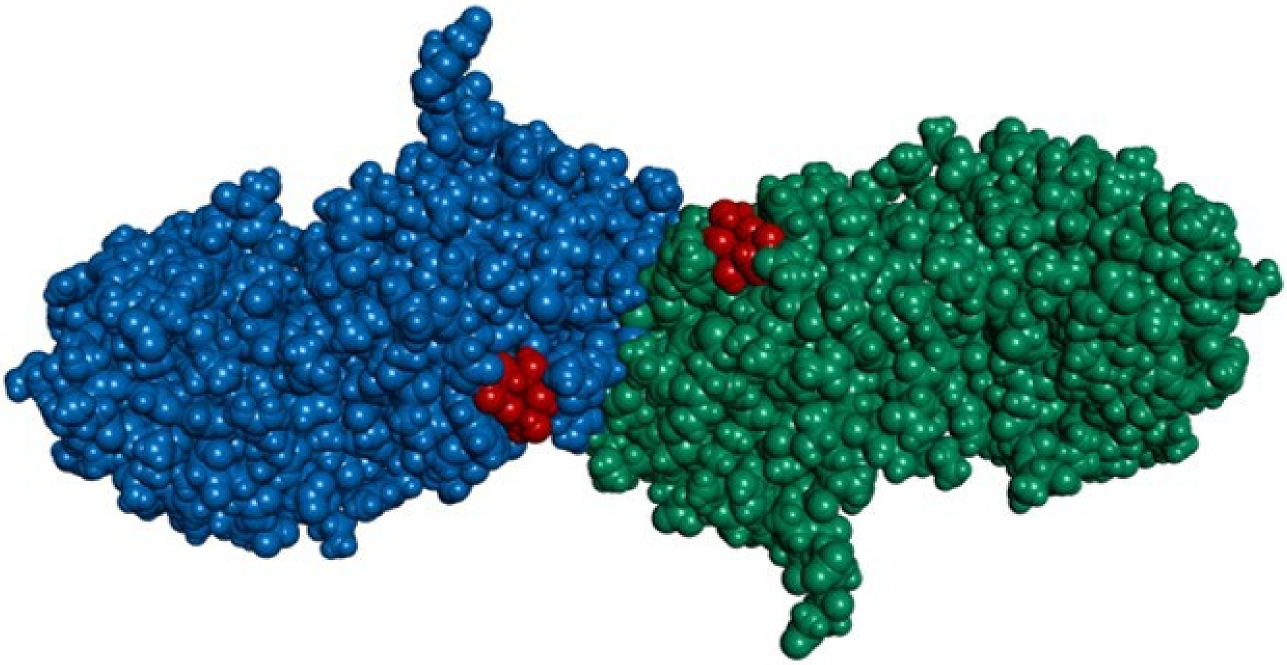

Figure S10. Structure of the SNAld dimer with D-galactose bound to 1α sites. Three-dimensional structural modeling was carried out on the Symmdock server from the monomer model provided by AlphaFold2. β-D-galactopyranose docking was performed with the Autodock 4.2, and the figure was generated using Discovery Studio 2021, as indicated in Materials and Methods. The atoms of the two monomers are represented by blue and green balls and the D-galactose molecules bound to the 1α sites are represented by red balls.
